# Whole Genome Sequencing Reveals How Plasticity and Genetic Differentiation Underlie Sympatric Morphs of Arctic Charr

**DOI:** 10.1111/mec.70085

**Published:** 2025-08-26

**Authors:** Khrystyna Kurta, Mariano Olivera Fedi, Kendall Baker, Tom Barker, Leah Catchpole, Claudio Ciofi, Arianna Cocco, Joanna Collins, Genevieve Diedericks, Maria Angela Diroma, Alex Durrant, Kjetil Hindar, Alessio Iannucci, Naomi Irish, Vanda Knitlhoffer, Linda Laikre, Henrique G. Leitão, Sacha Lucchini, Seanna McTaggart, Arnar Pálsson, Mats E. Pettersson, Nils Ryman, Sigurður S. Snorrason, Hannes Svardal, David Swarbreck, Robert M. Waterhouse, Christopher Watkins, Jonathan M. D. Wood, Han Xiao, Karim Gharbi, Zophonías O. Jónsson, Leif Andersson

**Affiliations:** ^1^ Department of Medical Biochemistry and Microbiology Uppsala University Uppsala Sweden; ^2^ Department of Animal Biosciences Swedish University of Agricultural Sciences Uppsala Sweden; ^3^ Earlham Institute Norwich UK; ^4^ Department of Biology University of Florence Sesto Fiorentino Italy; ^5^ Tree of Life Wellcome Sanger Institute Hinxton UK; ^6^ Department of Biology University of Antwerp Antwerp Belgium; ^7^ Norwegian Institute for Nature Research (NINA) Trondheim Norway; ^8^ Department of Zoology Stockholm University Stockholm Sweden; ^9^ Institute of Life‐ and Environmental Sciences University of Iceland Reykjavík Iceland; ^10^ Department of Ecology and Evolution University of Lausanne Lausanne Switzerland; ^11^ SIB Swiss Institute of Bioinformatics Lausanne Switzerland; ^12^ Department of Veterinary Integrative Biosciences, College of Veterinary Medicine and Biomedical Sciences Texas A&M University College Station Texas USA

**Keywords:** adaptation, Arctic charr, genetic differentiation, phenotypic plasticity, sympatric morphs, whole genome sequencing

## Abstract

Salmonids have a remarkable ability to form sympatric morphs after postglacial colonisation of freshwater lakes. These morphs often differ in morphology, feeding and spawning behaviour. Here, we explored the genetic basis of morph differentiation in Arctic charr (*n* = 283) by first establishing a high‐quality reference genome and then using this in whole genome sequencing of distinct morphs present in two Norwegian and two Icelandic lakes. The four lakes represent the spectrum of genetic differentiation between morphs from one lake with no genetic differentiation between morphs, implying phenotypic plasticity, to two lakes with locus‐specific genetic differentiation, implying incomplete reproductive isolation, and one lake with strong genome‐wide divergence consistent with complete reproductive isolation. As many as 12 putative inversions ranging from 0.45 to 3.25 Mbp in size segregated among the four morphs present in one lake, Thingvallavatn, and these contributed significantly to the genetic differentiation among morphs. None of the putative inversions were found in any of the other lakes, but there were cases of partial haplotype sharing in similar morph contrasts in other lakes. Our findings are consistent with a highly polygenic basis of morph differentiation with population‐specific selection on alleles linked to the development of similar morph phenotypes. The results support a model where morph differentiation is first established through phenotypic plasticity, leading to niche expansion and separation. This may be followed by gradual development of reproductive isolation, locus‐specific differentiation and eventually complete reproductive isolation and genome‐wide divergence.

## 1 Introduction

One of the major challenges in evolutionary biology is to understand how species diversify into genetically distinct populations, subspecies and ultimately separate species (Coyne and Orr 2004; Grant and Grant 2020). As the Arctic ice caps receded at the end of the last glacial epoch, many species of salmonids colonised different freshwater systems across the Northern hemisphere. Salmonid species belonging to five different genera (*Coregonus*, *Oncorhynchus*, *Prosopium*, *Salmo* and *Salvelinus*) are known for their ability to form genetically differentiated sympatric morphs that differ in morphology and/or feeding behaviour (reviewed by Salisbury and Ruzzante 2022). Considering that environmental factors are crucial drivers of sympatric differentiation (Corrigan et al. 2011), phenotypic plasticity has been regarded as a rapid response mechanism that could generate functional variation and foster adaptation (Fox et al. 2020; Skúlason et al. 2019). Under conditions where the environmental pressures remain consistent over generations, these plastic responses can facilitate the establishment of genetically differentiated morphs (Guðbrandsson et al. 2019). The presence of such sympatric morphs in freshwater systems across the Northern hemisphere provides an excellent opportunity to explore questions of fundamental importance in evolutionary biology, including the genetics of phenotypic differentiation, genetic parallelism and mechanisms underlying reproductive isolation.

The Arctic charr ( *Salvelinus alpinus* ) has a Holarctic distribution and is found both as permanent freshwater resident and anadromous populations (Johnson 1980; Smith et al. 2024). It has colonised thousands of lake systems across the Holarctic region and in alpine regions of the boreal zone (Brunner et al. 2001) and shows extensive phenotypic differentiation within and among lakes (see e.g., Adams and Maitland 2007; Klemetsen 2010). The co‐existence of two or more discrete phenotypes (or morphs) differing in traits such as body size, morphology, diet, habitat use and life‐history strategies of Arctic charr within a lake has repeatedly been reported, as reviewed by Salisbury and Ruzzante (2022). To name some well‐known examples, genetically distinct small and large morphs of Arctic charr occur in lakes in Labrador, Canada (Salisbury et al. 2020) and dwarf, small and large charr each occur in multiple lakes in northern Transbaikalia, Russia (Gordeeva et al. 2014). In Iceland, sympatric morphs differing in feeding ecology and habitat use have been described in several lakes (Brachmann et al. 2022; Gíslason et al. 1999) and, most notably, Lake Thingvallavatn, which contains four genetically and ecologically distinct morphs (Malmquist et al. 1985; Sandlund et al. 1992). Similar patterns have also been observed in Norway, where two (Hindar et al. 1986; Moccetti et al. 2019) to four genetically differentiated morphs co‐occur in multiple lakes (Østbye et al. 2020; Simonsen et al. 2017). Given the rich data on phenotypic and genetic differentiation among Arctic charr morphs, they represent an excellent model for investigating the genetic basis of sympatric divergence.

The presence of sympatric morphs of Arctic charr, suggestive of at least partial reproductive isolation, is well documented (Gíslason et al. 1999; Guðbrandsson et al. 2019; Jonsson and Hindar 1982). Furthermore, many studies suggest repeated independent evolution of the morphs in different lake systems, as morphs within the same lake are often more genetically related to each other than to similar morphs from other lakes (Alekseyev et al. 2002; Gíslason et al. 1999; Gordeeva et al. 2014; Salisbury et al. 2020). Though not all morphs show clear genetic divergence. In some cases, the differentiation of Arctic charr into distinct morphs might occur via plastic responses during development, for example, due to varying food resource utilisation, without specific genetic factors involved (Hindar and Jonsson 1993; Snorrason et al. 1994). However, the absence of genetic contributions to phenotypic differentiation can only be concluded after whole genome sequencing (WGS) has been performed because gene flow between sympatric populations can erase genetic differentiation at neutral loci. While other approaches have been successful in detecting genetic differences between some morphs, WGS has the power to uncover subtle, ‘cryptic differences’ that may not be detected with limited marker sets (Andersson et al. 2024). Furthermore, access to a high‐quality reference genome is a prerequisite for high‐resolution genomic, transcriptomic and epigenomic studies. However, omic studies in salmonids have been hampered by genome complexity due to an ancestral whole genome duplication that happened about 100 million years ago (Mya) (Macqueen and Johnston 2014). The varying degree of rediploidisation and presence of highly similar paralogs make it challenging to establish high‐quality genome assemblies and to accurately characterise many types of sequence variation. Previous studies on sympatric differentiation in Arctic charr have been based on restricted sets of genetic markers (microsatellites, AFLP—amplified fragment length polymorphisms and RADseq—restriction site associated DNA markers; Saha et al. 2024). Until recently, the only available reference genome for Arctic charr genomics was a genome assembly of a possible hybrid with a closely related species, the Northern Dolly Varden (*S. malma malma*: accession number GCA_002910315.2). However, recently two additional genome assemblies have been made public: (i) a scaffold‐level assembly of a Canadian Arctic charr subspecies ( *Salvelinus alpinus oquassa* ; accession number GCA_036784965.1), and (ii) an assembly representing a selectively bred line of Arctic charr ( *Salvelinus alpinus* ; accession number GCA_045679555.1).

In this study, we have performed whole‐genome sequencing of Arctic charr with the aim to close gaps in understanding the genetic basis of sympatric differentiation in Arctic charr. As a foundation for this analysis, we generated a high‐quality, chromosome‐level reference genome with annotated protein‐coding genes. We use this resource as a powerful tool for a population genomics project involving low‐pass, whole genome sequencing of 283 individuals representing different sympatric morphs from two Icelandic and two Norwegian lakes. A total of seven different morphs were included in the study: dwarf benthic (DB), large pelagic (LP), large generalist (LG), piscivorous (Pi), planktivorous (PL), large benthic (LB) and small benthic (SB). The specific objectives of this study were to: (i) characterise genome‐wide patterns of genetic differentiation among sympatric Arctic charr morphs across ecologically diverse lake systems; (ii) assess the extent of parallelism and divergence in morph differentiation across lakes; and (iii) identify genomic regions and sequence variants potentially involved in morph‐specific adaptation. By reanalysing the original samples from previous genetic studies in Norwegian charr (Hindar et al. 1986) alongside new samples from ongoing research in Iceland utilising ddRAD‐sequencing (Xiao et al. 2024) (see Table S1), this work provides a comprehensive and updated genome‐wide analysis of Arctic charr morph differentiation.

## 2 Materials and Methods

### 2.1 Reference Genome Assembly and Annotation

A large benthivorous charr from Lake Thingvallavatn was chosen for genome and transcriptome sequencing as part of the Pilot Project of the European Reference Genome Atlas (ERGA) initiative (Mc Cartney et al. 2024). Data generation, genome assembly and gene annotation were carried out at the Earlham Institute (United Kingdom), with the exception of Hi‐C library preparation (University of Antwerp, Belgium), Hi‐C sequencing (University of Florence, Italy) and genome curation post‐assembly (Wellcome Sanger Institute, United Kingdom). Full details are available in Methods S1.

### 2.2 Samples for Whole Genome Resequencing

**FIGURE 1:**
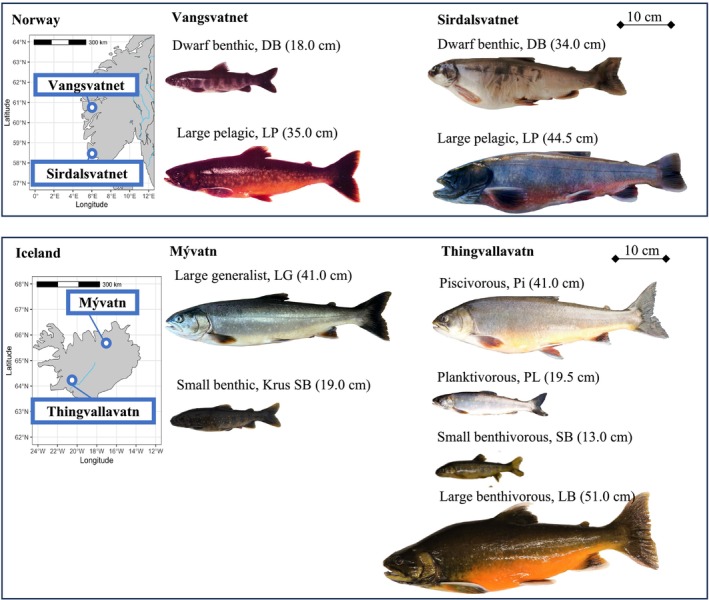
Geographical location and phenotypic differentiation of Arctic charr ( *Salvelinus alpinus* ) morphs from Norway (Vangsvatnet and Sirdalsvatnet) and Iceland (Mývatn and Thingvallavatn). Fork lengths of the fish are shown in brackets next to the morph name. The reference genome generated in this study came from the large benthivorous charr (LB‐charr) shown in the bottom right corner. Photo credits: Tormod A. Schei (Vangsvatnet), Ragnvald Andersen (Sirdalsvatnet), Gylfi Yngvason and Árni Einarsson (LG, Mývatn); Sigurður S. Snorrason and Zophonías O. Jónsson (SB, Mývatn); Sigurður S. Snorrason, Zophonías O. Jónsson and Arnar Palsoon (Thingvallavatn).

Arctic charr samples for WGS were obtained from two lakes in Iceland: Thingvallavatn and Mývatn and from two lakes in Norway: Vangsvatnet and Sirdalsvatnet (Figure 1). Samples from the two Norwegian lakes were from a previously published study (Hindar et al. 1986).

The four charr morphs of Lake Thingvallavatn were sampled at the respective spawning grounds using gillnets of various mesh sizes. LB charr were sampled at Ólafsdráttur. PL, Pi and SB charr were sampled on both sides of the Mjóanes peninsula. The sampling effort and DNA extraction are described in detail in Xiao et al. (2024).

Samples of the large generalist morph (LG) from Lake Mývatn were caught in gillnets in the main basin of the lake as part of regular fisheries surveys by the Icelandic Marine and Freshwater Research Institute and kindly provided by Guðni Guðbergsson. Sampling for the SB morph (locally known as ‘Krús’) was conducted at Kálfaströnd, in an area of cold‐water springs (N 65°33.841 W 16°56.681). The fish were caught by electrofishing, anaesthetised using phenoxyethanol at low concentration and measured, weighed and photographed. Small clippings from the caudal fin were stored in ethanol, and the fish were then released. Many fish from Kálfaströnd were 0+ and 1+ juveniles that could not be assigned to morph based on morphology. As the cold spring area is a known habitat harbouring the SB morph, all the fish caught at Kálfaströnd were pre‐assigned to that morph. Morph assignment was later revised based on the photographs and genetic data (Figure S3).

Detailed information on the number of samples from each morph at each sample location is presented in Table S1.

### 2.3 DNA Extraction and Sequencing

Extracted genomic DNA was available for the Icelandic samples (Xiao et al. 2024). Briefly, ethanol‐preserved tissue from fin clips or muscle was rehydrated and digested with proteinase K, followed by DNA isolation using standard phenol/chloroform extraction. Muscle tissue preserved in 99% ethanol was used for DNA extraction from the Norwegian samples. Genomic DNA was extracted using the Monarch Genomic DNA Purification Kit (New England Biolabs). The purity of the DNA was assessed with Nanodrop 1000, and the DNA quantity was assessed using the Qubit dsDNA BR Assay Kit (Thermo Fisher Scientific) and a Tecan microplate reader. The DNA was diluted to a final concentration of 10 ng/μL for library preparation. Custom Tn5‐based libraries (Picelli et al. 2014) with a target size of 350 bp were constructed for each DNA sample (*n* = 283). All libraries were individually barcoded and pooled. The TapeStation D1000 (Agilent Technologies) was used to visualise library size. The library pool was sequenced on two Illumina NovaSeq S4 lanes.

The Illumina raw reads were mapped to the Arctic charr assembly (ENA Project PRJEB76174), developed as part of this study. Mapping was done using ‘bwa mem ‐M’ v0.7.17 (Li 2013). The resulting alignments were sorted using Samtools v1.10 (http://www.htslib.org/) and finally processed with MarkDuplicates from PicardTools v1.92 (https://broadinstitute.github.io/picard/). The average sequencing depth per individual was 2.1 ± 0.9X (range: 0.22–6.11X). The majority of individuals (*n* = 264) had genome‐wide average coverage above 1.19X (the mean minus one standard deviation; Figure S4).

### 2.4 Genotype Likelihood Estimation and Quality Control

Due to the limitations of the low sequencing coverage approach for genotype calling, we estimated genotype likelihoods using ANGSD v0.933 (Korneliussen et al. 2014). The genotype likelihoods were used to estimate allele frequencies for each morph (*n* = 22–40, Table S1) or population (*n* = 52–111, Table S1). Given the salmonid‐specific whole‐genome duplication, we recognise that retained homeologous sequences may lead to ambiguous read mapping and inflated variant calling. To mitigate this, and minimise potential mis‐mapping to duplicated or repetitive genomic regions, we applied stringent filtering criteria in ANGSD: ‘‐minMapQ30 ‐uniqueOnly 1 ‐remove_bads 1 – only_proper_pairs 0 ‐trim 0 ‐GL 2’.

We used ‘–doMajorMinor 4 –minMaf 0.05’ options to generate the list of positions with minor allele frequency (MAF) > 5%. To generate the high‐confidence variants for the analysis, we supplied ‘‐SNP_pval 1e‐6’ to ANGSD. The total set of variant positions across the entire dataset was ~12 million, of which 1.5 million were on unplaced scaffolds. The list of polymorphic positions was supplied as ‘‐sites’ argument for genotype likelihood, population‐ or morph‐wise allele frequencies estimation and genome‐wide contrast (GWC) study. We performed principal component analysis (PCA), estimated ancestry proportions and calculated pairwise Nei distances based on a SNP dataset that was pruned to reduce linkage disequilibrium (LD). LD pruning was performed with PLINK v1.9 using a window size of 100 SNPs, a step size of 5 SNPs and a VIF threshold of 2 (indep 100 5 2). The resulting set of pruned SNPs per population was ~1 million SNPs for all lakes combined, 0.95 million SNPs for Sirdalsvatnet, 0.91 million SNPs for Vangsvatnet, 0.69 million SNPs for Mývatn and 0.70 million SNPs for Thingvallavatn. Nucleotide diversity calculation used all observed positions. All data were obtained for 40 scaffolds, while unplaced scaffolds were summarised into a NA scaffold.

### 2.5 Analysis of Population Structure

To examine population structure and genetic divergence, we ran PCA using PCAngsd v1.11 (Meisner and Albrechtsen 2018) for the datasets across and within lakes. The built‐in R function ‘eigen()’ was used to extract eigenvalues and eigenvectors, ‘ggplot2’ package was used to plot the data.

We calculated individual admixture proportions using PCAngsd v1.11 (Meisner and Albrechtsen 2018) and NGSadmix v. 32 (Skotte et al. 2013) modules from ANGSD. PCAngsd was used for all lakes combined and determines the optimal number of clusters (K) empirically based on the major principal component (PC) loadings. In contrast, NGSadmix was applied within each lake separately, with a predefined K. To identify the best‐supported number of clusters (K), we evaluated log‐likelihoods and Frobenius errors across a range of K values (1 to 10) following Meisner and Albrechtsen (2018). The optimal K was determined as the value that maximised the log‐likelihood and minimised the Frobenius error.

A neighbour‐joining (NJ) genetic distance tree of samples from a covariance matrix was derived based on individual allele frequencies using the ‘‐tree’ flag in PCAngsd (Meisner and Albrechtsen 2018). The NJ tree visualisation was done using an online tool iTOL (https://itol.embl.de/; Ciccarelli et al. 2006).

### 2.6 Genetic Diversity

To characterise nucleotide diversity for each sampling location and morph, we calculated the average number of pairwise differences between sequences (θ). All θ were calculated based on allele frequencies generated without MAF cut‐offs because MAF cut‐offs introduce bias in population‐based frequency diversity estimates. The following command, implemented in ANGSD v.0.933 (Korneliussen et al. 2014), was used to estimate allele frequencies for each sample group originating from the same lake or separately from each morph within a lake. This approach follows the recommended workflow described in the published pipeline https://github.com/clairemerot/angsd_pipeline.

angsd ‐P $NB_CPU ‐underFlowProtect 1 ‐dosaf 1 ‐GL 2 ‐doMajorMinor 1 ‐doCounts 1 ‐anc $REFGENOME ‐fai $REF_INDEXED ‐rf $REGION ‐remove_bads 1 ‐minMapQ 30 ‐minQ 20 ‐minInd $MIN_IND ‐setMaxDepth $MAX_DEPTH ‐setMinDepthInd 1 ‐bam $POP ‐out $OUT.

The filters above select sites with high‐quality sequencing depth and alignment, and to ensure the selected sites have sequence information in at least 50% (or 90% if sample size below 12) of the individuals per population (*‐minInd*). Maximum depth was set to 3× the expected coverage to remove repeated regions, following the recommendation of Mérot et al. (2021). After that, we used the ANGSD realSFS command to calculate a two‐dimensional folded site frequency spectrum (2D‐SFS). This was used as a prior to calculate diversity statistics with the saf2theta command. Nucleotide diversity (θ) was calculated in 20 kb non‐overlapping windows. Obtained values were averaged per population and divided by the number of sites per window to recover an unbiased estimate of nucleotide diversity as described in (Enbody et al. 2021).

Finally, population differences were assessed using pairwise *F*_ST_. The 2D‐SFS, obtained using the filters and the script https://github.com/clairemerot/angsd_pipeline/blob/master/01_scripts/07_fst_by_group.sh, was used as a prior to estimate *F*_ST_ statistics with the ANGSD realSFS fst index command. To perform a sliding window analysis, *F*_ST_ values were computed with a window size of 20 kb and a step size of 10 kb using the realSFS fst stats2 command, with the options ‐win 20,000 and ‐step 10,000. Known polymorphic sites were provided using the ‘‐sites’ option based on a filtered SNP set (as described above, MAF > 0.05, SNP *p*‐value < 1e‐6). The number of SNPs retained for analysis ranged from approximately 9.6 to 12.2 million across lake comparisons.

In the case of pairwise *F*_ST_ estimates between morphs within each lake, scaffolds without putative inversions (e.g., scaffolds 2, 6, 7, 10 and 11) were used with ~2.0 to ~11.4 million sites between morph comparisons. The obtained *F*_ST_ values for each population or morph pair were averaged across the scaffolds.

### 2.7 Genome Wide Screen for Genetic Differentiation

To identify genomic regions showing genetic differentiation between populations, we performed genome‐wide contrast (GWC) analysis. Specifically, we used the ‘‐doAsso 1’ function in ANGSD, which performs a likelihood ratio test for allele frequency differences between groups (morphs) (Kim et al. 2011). This association study is based on individual genotype likelihoods and takes all uncertainty of the generated data into account (Korneliussen et al. 2014). The method used compares allele frequencies between all pairs of morphs within a lake. The genome‐wide significance threshold was calculated using a Bonferroni correction with a significance level at *α =* 10^−3^ and *α =* 10^−8^ using the following formula: Bonferroni = −log10(*α*/N), where N is the number of SNPs.

### 2.8 Analysis of Putative Structural Variants

Candidate inversion regions were identified as clusters of highly differentiated SNPs forming contiguous block‐like patterns along scaffolds with steep declines in differentiation at the block edges, as described in Han et al. (2020). Within these regions, we quantified linkage disequilibrium (LD) using ngsLD/1.1.1 between SNP pairs (Pearson *r* ^*2*^) to support the presence of extended haplotype blocks.

To further explore the inversion haplotype structure, we estimated genotype posterior probabilities using the ‘‐doGeno’ and ‘‐doPost’ options in ANGSD. For each sample and site, we summed genotype probabilities corresponding to the reference and alternative alleles and identified the most common allele in a predefined reference group. Only sites with high‐confidence posterior probabilities and significant differentiation (diagnostic markers, SNPs exceeding a Bonferroni‐corrected threshold of α = 10^−8^) were retained. We then performed principal component analysis (PCA) on SNPs located within each candidate inversion to visualise genotype clustering. Based on the PCA and genotype likelihood profiles, individuals were grouped into three genotype classes: homozygous for the major allele, heterozygous and homozygous for the minor allele. Finally, we compared nucleotide diversity (θ) among these genotype groups using the Wilcoxon rank‐sum test to assess differences in genetic diversity associated with inversion genotype status using R software (R Core Team 2019).

### 2.9 Analysis of Haplotype Sharing Outside Putative Structural Variants

To investigate whether similar patterns of genetic differentiation existed outside the putative structural variants among the same morphs from different lakes, we generated heatmaps of allele frequencies at diagnostic markers (SNPs exceeding a Bonferroni‐corrected threshold of α = 10^−8^). Regions displaying similar allele frequency patterns across morphs and lakes, particularly those spanning larger genomic intervals and containing clusters of closely spaced diagnostic SNPs with high LD, were defined as *shared haplotype‐like block* (hereafter referred to as *shared haplotypes* for simplicity). To further explore the shared haplotype structure, we estimated genotype posterior probabilities as described above. To validate the presence of shared haplotypes, individuals clustered as homozygous major and homozygous minor were then used for the PCA and NJ genetic distances tree using PCAngsd (Meisner and Albrechtsen 2018).

### 2.10 Functional Annotation of Gene Models

The gene models in the reference genome (described above) initially only included gene IDs and lacked gene symbols and descriptions. To address this and enhance interpretations of the results, we utilised the functional gene annotation pipeline provided by the National Bioinformatics Infrastructure Sweden (NBIS, Binzer‐Panchal et al. 2021) to obtain the missing information. Briefly, the nucleotide sequence of each annotated gene was extracted from the genome sequence in FASTA format using the gene coordinates from the GFF annotation file. The nucleotide sequence per gene was then translated into an amino acid sequence using Another Gtf/Gff Analysis Toolkit (AGAT v.1.3.2) (Dainat et al. 2024). The amino acid sequence was compared against a reference protein database to infer which protein corresponds to each gene using BLASTp v.2.15.0+ (Altschul et al. 1990) with blast e‐value = 1e‐6. As a reference, we used the reviewed Vertebrates protein database available from UniProtKB/SwissProt in July 2021 (version 2021_03). Gene function annotation was conducted using STRING v12 (https://string‐db.org/), with *Homo sapiens* selected as the reference gene database for the analysis. Finally, we annotated the nearest gene (within ±5 kb) and assessed the variant effects (such as missense, synonymous, upstream, downstream or intergenic) for the most differentiated SNPs (*p* < 1 × 10^−15^) identified in each putative inversion or shared region of divergences using SnpEff v.4.1 (Cingolani et al. 2012).

## 3 Results

### 3.1 Genome Assembly Overview

We assembled the genome of a male large benthic Arctic charr sampled from Lake Thingvallavatn in Iceland using 8.7 M Pacific Biosciences high‐fidelity (HiFi) reads with a mean length of 18 kb, generating approximately 52‐fold coverage of the haploid genome (Table S2). Primary assembly contigs were scaffolded with Hi‐C chromosome conformation data from 386 M Illumina short reads, providing approximately 38‐fold coverage. The final, manually curated assembly has a total length of 2.99 Gb in 5789 sequence scaffolds with a scaffold N50 of 53.2 Mb (Table 1). A total of 40 scaffolds were assembled to chromosome level ranging from 91.8 to 8.2 Mb in size. Another 17 scaffolds were assigned to chromosomes but not placed within the main scaffolds. The remaining 5732 scaffolds remained unplaced. The total span of chromosome‐level scaffolds and scaffolds assigned to chromosomes was 2.41 Gb. With the exception of BUSCO metrics showing an excess of duplicated genes consistent with tetraploid ancestry, assembly metrics exceeded Earth BioGenome Project (EBP, https://www.earthbiogenome.org/report‐on‐assembly‐standards) standards (Lawniczak et al. 2022) and were broadly similar to those of published assemblies derived from other salmonid species using equivalent data types, including HiC, for example, (Christensen et al. 2024; De‐Kayne et al. 2020; Lawniczak et al. 2022; Rondeau et al. 2023). We also found extensive synteny between our assembly and the high‐density linkage map of Canadian Arctic charr (Figure S1) as well as the two chromosome‐level assemblies currently associated with Arctic charr (Figure S2 and Tables S3 and S4). In total, we identified 47,703 protein‐coding genes and 12,061 transposable element genes with high confidence. Our population genomic analyses are based on the scaffold‐level assembly of the genome. As a quality control, we mapped all short reads from the population genomics analysis (next section) to the reference assembly (Figure S5). This analysis revealed a generally even sequence coverage across the genome and we did not detect any whole chromosomes as collapsed duplicated copies. However, we noticed localised regions of elevated coverage on scaffolds 2, 13, 17, 22, 26 and 33 most likely due to collapsed repeats or duplications. This needs to be taken into account when interpreting the population genomics data.

**TABLE 1:** Genome assembly and annotation statistics for *Salvelinus alpinus* (fSalAlp1.1.hap1.cur.20231016).

Assembly metrics	
Span (bp)	2,996,861,510
Number of scaffolds	5,789
Scaffold N50 length (bp)	53,120,343
Number of contigs	10,412
Contig N50 length (bp)	859,180
Consensus quality (QV)	56.34
*k*‐mer completeness	95.28%
**BUSCO analysis**	
Complete (%)	97.2
Complete and single copy (%)	51.3
Complete and duplicated (%)	45.9
**Annotation**	
Number of protein‐coding genes	47,703
Number of protein‐coding transcripts	128,882
Number of transposable element genes	12,061
Number of transposable element transcripts	13,030

### 3.2 Genome‐Wide Differentiation and Population Structure

**FIGURE 2:**
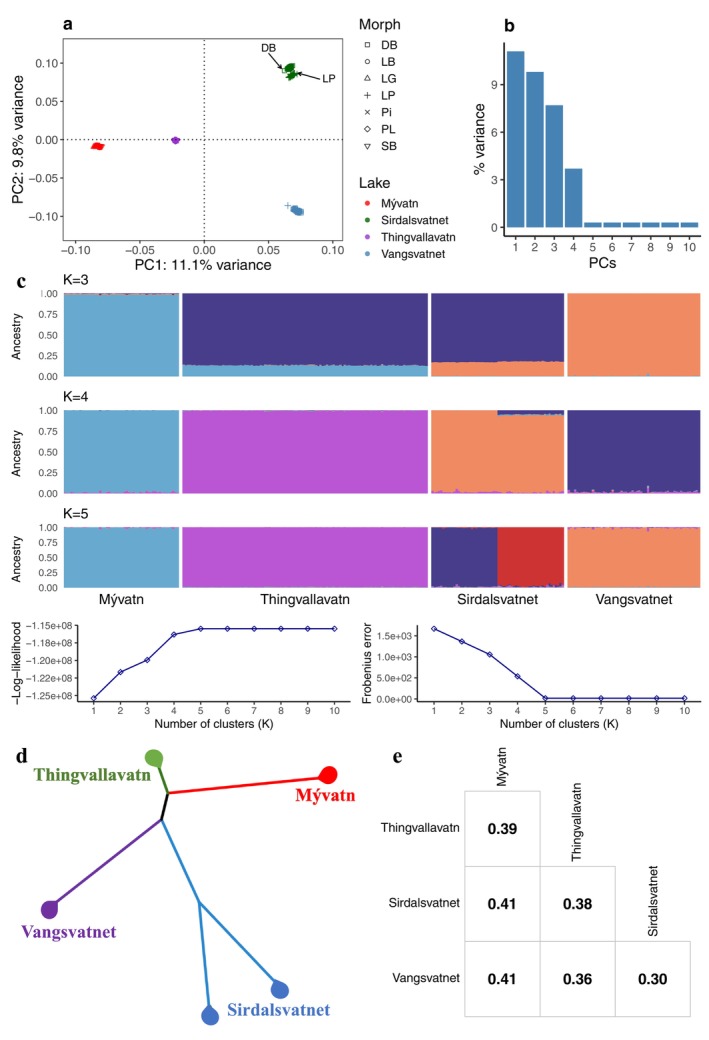
Genetic differentiation in Arctic charr among lakes and morphs. PCA, Admixture and Neighbour‐joining tree analyses generated in PCAngsd based on a list of LD‐pruned ~1 million SNPs (MAF > 0.05). Scores of individuals along principal components (a) PC1 and PC2 and (b) proportion of explained variance across 10 principal components. (c) Ancestry proportions with ‐Log‐likelihoods and Frobenius error for 1–10 clusters. (d) Unrooted Neighbour‐joining tree and (e) pairwise *Fst* values illustrating genetic differentiation between lakes.

Low pass sequencing was carried out on Arctic charr sampled from four lakes: Thingvallavatn and Mývatn in Iceland, and Sirdalsvatnet and Vangsvatnet in Norway, representing 2–4 genetically and ecologically distinct morphs per lake (Figure 1; Table S1). Sequence coverage per individual was on average 2.1 ± 0.9X (range: 0.22–6.11X; Figure S4). Inference of population structure was done using genotype likelihoods from the ANGSD software (Korneliussen et al. 2014). As expected from the geographic distances between these lakes and the topography of their out‐flowing rivers, we found strong genetic differentiation between lakes (Figure 2a–c). Furthermore, and consistent with earlier studies (Gíslason 1998; Hindar et al. 1986; Kapralova et al. 2011; Xiao et al. 2024), different morphs from the same lake clustered tightly along the first two principal component (PC) axes (Figure 2a,b). Admixture analysis with PCAngsd gave the highest support at *K* = 5 (Figure 2c) and, as in the case of the PCA, revealed genetic separation by lakes, but also two distinct clusters in Lake Sirdalsvatnet, highlighting strong genetic differentiation between the dwarf benthic and large pelagic morphs in this lake. Consistently, the neighbour‐joining tree showed four clusters separating the four lakes with an additional split in Sirdalsvatnet (Figure 2d). Pairwise *Fst* values between population pairs (i.e., lakes) ranged from 0.30 ± 0.20 to 0.41 ± 0.22 (Figure 2e), further indicating substantial genetic differentiation among lakes. Notably, nucleotide diversity was higher in the Norwegian lakes (0.20%–0.22%) compared to the Icelandic lakes (0.16%–0.17%; Table S5).

### 3.3 Genetic Differentiation and Population Structure Between Morphs Within Each Lake

**FIGURE 3:**
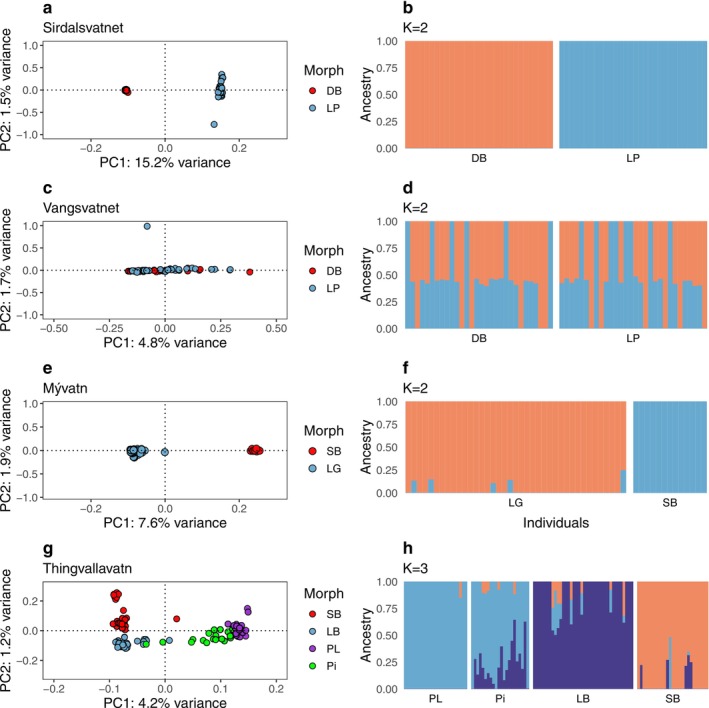
Genetic differentiation among Arctic charr morphs in two Norwegian and two Icelandic lakes. Scores of individuals along PC1 and PC2 with the genetic variance explained from PCA (left), and ancestry proportions (right) for each lake. PC scores were generated using PCAngsd, and individual ancestry proportions were estimated with NGSadmix based on a list of SNPs pruned for linkage disequilibrium and MAF > 0.05 in every lake. The numbers of SNPs used per population: (a, b) Sirdalsvatnet (0.95 million SNPs), (c, d) Vangsvatnet (0.91 million SNPs), (e, f) Mývatn (0.69 million SNPs) and (g, h) Thingvallavatn (0.70 million SNPs). Colours on PCA plots indicating the sympatric morphs of Arctic charr for Sirdalsvatnet and Vangsvatnet: Dwarf benthic (DB) and Large pelagic (LP); for Mývatn, Large generalist (LG) and Small benthic (Krús, SB), and Thingvallavatn, Piscivorous (Pi), Planktivorous (PL), Large benthivorous (LB) and Small benthivorous (SB).

First, we compared dwarf benthic and large pelagic morphs in the Norwegian lakes Sirdalsvatnet and Vangsvatnet and found high genome‐wide divergence between the morphs from Sirdalsvatnet (Figure 3a,b). Admixture analysis showed the highest support at *K* = 2, separating the two morphs in Sirdalsvatnet (Figure 3b). The average *Fst* value between the two morphs in Sirdalsvatnet was 0.19 ± 0.19 (mean ± s.d). In sharp contrast, the corresponding morphs (dwarf benthic and large pelagic) in Vangsvatnet were not genetically differentiated (Figure 3c,d), and average *Fst* was as low as 0.01 ± 0.00 (mean ± s.d). The genome‐wide contrast (GWC) detected genome‐wide differentiation between the Sirdalsvatnet morphs (Figure S6a, Table S6), consistent with strong reproductive isolation and genetic differentiation at both neutral and adaptive loci. Genetic differentiation may have occurred sympatrically or, alternatively, the two populations may have colonised the lake separately. As expected, no significant differentiation was found in a GWC between the two morphs in Lake Vangsvatnet (Figure S6b). This could indicate that the two morphs in Vangsvatnet are not reproductively isolated.

In Lake Mývatn, we compared fish classified as small benthic and large generalist. PCA showed two distinct genetic groups separated along the PC1 axis that explained 7.6% of the variance (Figure 3e). Additionally, admixture analysis suggested the presence of *K* = 2 distinct genetic backgrounds with little evidence of shared genetic variation, as indicated by two LG individuals exhibiting ~10%–24% admixture from the SB group (Figure 3f). The average *Fst* ± s.d. (0.03 ± 0.05) indicates a moderate level of genetic differentiation between morphs in Mývatn. A GWC contrasting these genetically classified morphs detected single nucleotide polymorphisms (SNPs, *n* = 198) exceeding the Bonferroni‐adjusted significance threshold across 20 scaffolds (Figure S6c, Table S6).

Lake Thingvallavatn harbours four morphs, and the first PC (4.2% of variance) separated the benthic morphs from the planktivorous morph and the majority of piscivorous individuals, while PC2 (1.2% of variance) separated the small and large benthivorous morphs (Figure 3g). The result confirms previously published findings based on allozyme loci (Magnusson and Ferguson 1987) and microsatellites (Gíslason 1998). As previously reported (Guðbrandsson et al. 2019), most piscivorous individuals grouped with or close to the planktivorous charr cluster; however, several individuals fell in between the large benthivorous and piscivorous/planktivorous clusters. Estimated admixture proportions with PCAngsd suggested *K* = 2 genetic backgrounds, likely corresponding to the broad separation of benthivorous and piscivorous/planktivorous morphs, as revealed by PC1. However, NGSadmix detected an additional layer of genetic structure (*K* = 3, Figure S7a,b), consistent with the separation of the small and large benthivorous morphs (captured by PC2) with evidence of shared genetic variation between the four morphs (Figure 3h). Notably, the *Fst* values (Figure S7c) for the benthivorous versus planktivorous morph pairs suggested moderate differentiation (*Fst* in the range 0.06 ± 0.03 to 0.08 ± 0.07), while the piscivorous/planktivorous contrast exhibited minimal differentiation (*Fst* = 0.02 ± 0.02). *Fst* between large/small benthivorous morphs equalled 0.04 ± 0.06. Consistent with these data, the GWC analysis identified multiple genomic regions showing significant genetic differentiation, defined by clusters of SNPs (from 563 to 9861; Table S6) exceeding the genome‐wide significance threshold (*p* < 1 × 10^−10^), between all morph pairs from Thingvallavatn (Table S6, Figure S6d–i) except for the piscivorous/planktivorous contrast, where a single significant SNP was found on scaffold 15 (Figure S6i).

### 3.4 Genetic Differentiation at Putative Inversions Present in Lake Thingvallavatn

**FIGURE 4:**
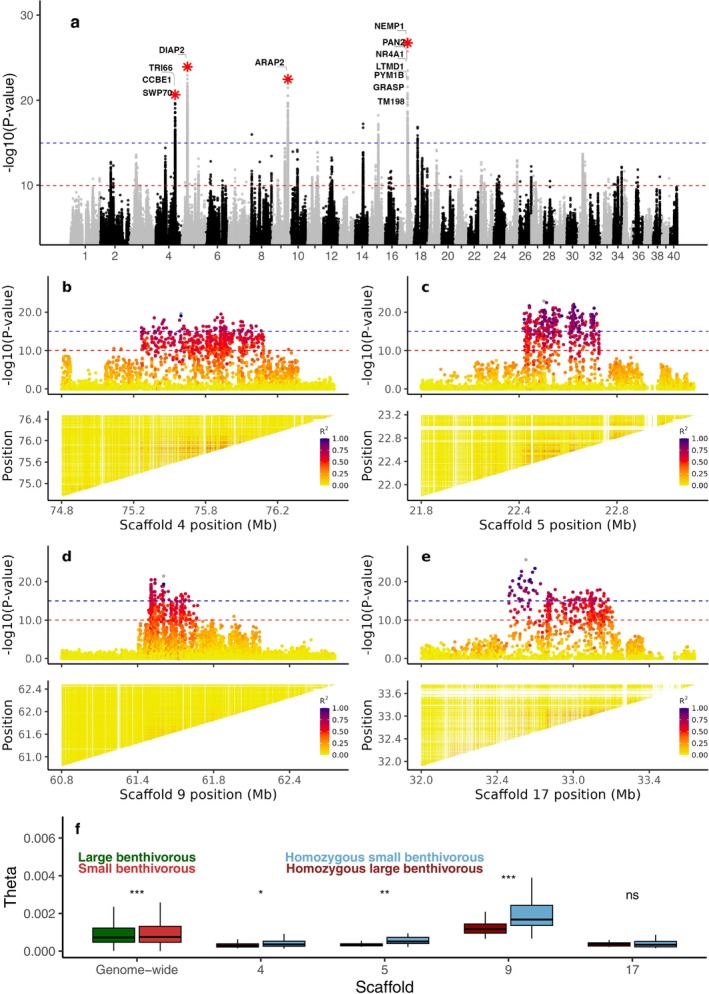
Genetic differentiation between small and large benthivorous morphs of Arctic charr in Thingvallavatn. (a) Genome scan based on estimated allele frequencies for individual SNPs. Gene annotations are shown for the top 0.001% of significant SNPs at putative inversions marked by stars. (b–e) Zoom‐in profile of four putative inversion regions in (a). Top panel: Linkage disequilibrium (LD), measured as *r* ^2^, between the top significantly associated SNP and surrounding variants; bottom panel: Pairwise LD (*r* ^2^) among all genotypes within each region, including the following genomic regions (b) Scaffold 4: 75.25–76.13 Mb, (c) Scaffold 5: 22.30–22.75 Mb, (d) Scaffold 9: 61.30–62.11 Mb and (e) Scaffold 17: 32.45–33.20 Mb. The horizontal red and blue lines indicate Bonferroni corrected significance thresholds with significance levels *α =* 10^−3^ and *α =* 10^−8^, respectively, after taking into account that 10^7^ SNPs were used. (f) Wilcoxon rank test of theta (θ, nucleotide diversity) distributions across the genome and within putative inversion regions. Significance levels: *p < 0.05, **p < 0.01, ***p < 0.001, ns indicates *p* > 0.05.

We next investigated the genomic regions that differentiate the two benthivorous morphs (LB and SB) in Lake Thingvallavatn. Highly differentiated SNPs above the Bonferroni threshold (*p* < 1 × 10^−10^) were detected across 33 scaffolds and unplaced scaffolds (Figure 4a, Table S6). Notably, four of the major outlier loci involved large genomic regions spanning 0.88 Mb on scaffold 4 (489 SNPs with *p* < 1 × 10^−10^), 0.45 Mb on scaffold 5 (409 SNPs), 0.81 Mb on scaffold 9 (277 SNPs) and 0.75 Mb on scaffold 17 (273 SNPs) (Figure 4b–e). A closer look at these loci revealed a block‐like pattern with many SNPs showing strong genetic differentiation and with sharp drops in differentiation at each end of the block, a pattern consistent with the presence of inversions (Han et al. 2020). However, we note that similar patterns could also occur in the absence of inversions in regions with suppressed recombination combined with natural selection that maintains favourable haplotype combinations. Strong linkage disequilibrium (LD) among SNPs within inversions was restricted to a subset of SNPs (*p* < 1 × 10^−10^) with the top outlier SNP, particularly on scaffolds: 4 (*r* ^2^ mean ± SD: 0.5 ± 0.07), 5 (*r* ^2^ mean ± SD: 0.5 ± 0.09), 9 (*r* ^2^ mean ± SD: 0.5 ± 0.08) and 17 (*r* ^2^ mean ± SD: 0.4 ± 0.9) (Figure 4b–e, Table S5). These putative inversions contain altogether 64 genes: 30 on scaffold 4, 1 on scaffold 5, 1 on scaffold 9 and 32 on scaffold 17 (Table S8). A summary of the most differentiated SNPs (*p* < 1 × 10^−15^) for each region, including their nearest gene and relative position to genes (e.g., nonsynonymous, synonymous, upstream, downstream or intergenic) as determined by snpEff are presented in Table S9.

We calculated nucleotide diversity (θ) for each haplotype within the four putative inversions to assess levels of genetic variation that reflect their evolutionary history. First, we determined the genotype at each locus (Table S10) and calculated nucleotide diversity for individuals homozygous for either the small or the large benthivorous‐associated inversion haplotype (Figure 4f). While the genome‐wide nucleotide diversity was very similar in the two morphs (dwarf: 0.15%; large benthic: 0.14%), the average θ values within the putative inversions at scaffolds 4, 5 and 9 were significantly higher (Wilcoxon test, *p* < 0.01) in the small (from 0.06% to 0.21%) than in the large benthivorous morph (from 0.04% to 0.13%). In contrast, within the putative inversion on scaffold 17, nucleotide diversity was similar between the two benthivorous morphs, with an average of 0.04% in both cases (Figure 4f, Table S11). We further explored the patterns of genotypes over these putative inversions, using only diagnostic markers (*p* < 1 × 10^−15^) among morphs from Thingvallavatn and the other lakes (Figure S8). This analysis illustrates the striking genetic differentiation between small and large benthivorous morphs of Thingvallavatn at these loci. These distinct patterns were further supported by local PCA conducted on all SNPs within each putative inversion region, with the exception of scaffold 17, where the haplotype structure was less clearly resolved (Figure S9). Furthermore, the large benthivorous haplotypes at scaffolds 4, 5 and 17 were strikingly similar to the haplotypes dominating among the pelagic morphs in the lake. We also explored the possibility that these putative inversions might segregate in any of the other three lakes included in this study, but we found no evidence for a similar pattern involving the entire regions (Figure S8). However, there was an interesting pattern at two relatively large fragments (61,387,699‐61,393,705 bp; and 61,501,590‐61,615,879 bp) within the putative inversion region on scaffold 9: 61.3–62.1 Mb, in which the small benthic morphs from Thingvallavatn (Iceland) and Sirdalsvatnet (Norway) shared haplotypes (Figure S8), whereas the planktivorous morph from Sirdalsvatnet shared the haplotypes of the large benthivorous morph from Thingvallavatn. This haplotype sharing likely represents ancestral polymorphisms that may have contributed to morph differentiation in many lake systems. In Thingvallavatn, alleles at these ancestral loci became part of the putative inversion.

**FIGURE 5:**
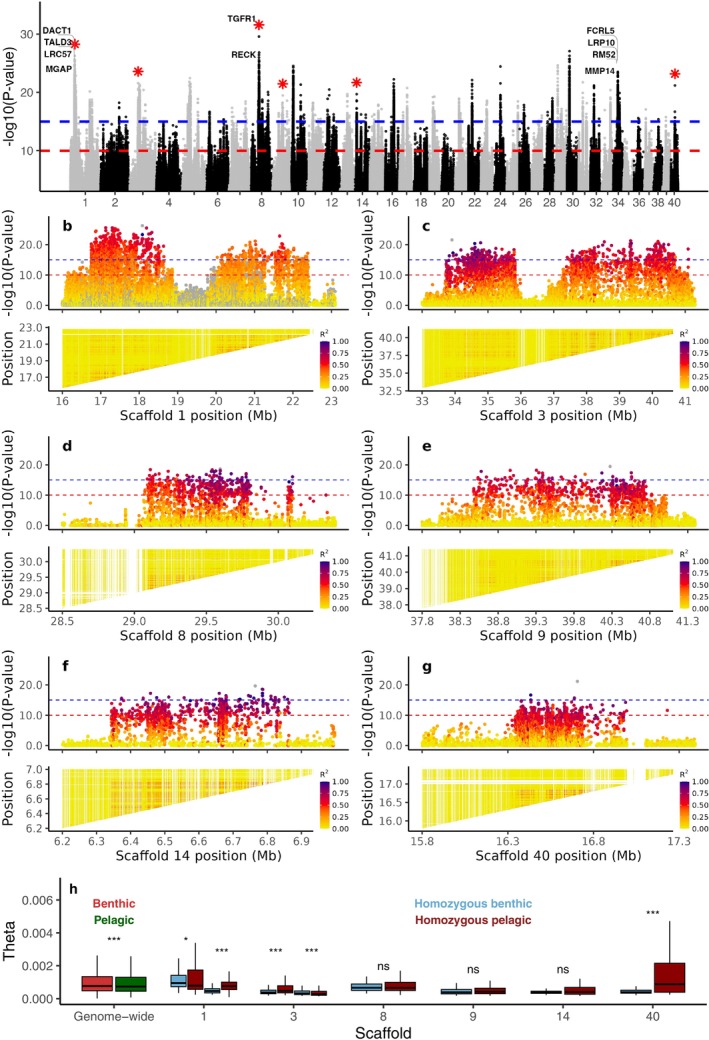
Genetic differentiation between benthic (large and small benthivorous) and pelagic (piscivorous and planktivorous) Arctic charr morphs in Thingvallavatn. (a) Genome scan based on estimated allele frequencies for individual SNPs. Gene annotations are shown for the top 0.001% of significant SNPs at putative inversions marked by stars and at scaffold 34. (b–g) Zoom‐in profile of four putative inversion regions in (a). Top panel: Linkage disequilibrium (LD), measured as *R* ^2^, between the top significantly associated SNP and surrounding variants; bottom panel: Pairwise LD (*r* ^2^) among all genotypes within each region, including the following genomic regions, including (b) Scaffold 1: 16.30–18.60 Mb and 19.50–22.20 Mb, (c) Scaffold 3: 33.50–35.80 Mb and 37.35–40.60 Mb, (d) Scaffold 8: 29.05–29.83 Mb, (e) Scaffold 9: 38.40–40.80 Mb, (f) Scaffold 14: 6.33–6.87 Mb and (g) Scaffold 40: 16.25–17.01 Mb. The horizontal red and blue lines indicate Bonferroni corrected significance thresholds with significance levels *α =* 10^−3^ and *α =* 10^−8^, respectively, after taking into account that 10^7^ SNPs were used. (h) Wilcoxon rank test of theta (θ, nucleotide diversity) distributions across the genome and within putative inversion regions. Significance levels: *p < 0.05, **p < 0.01, ***p < 0.001, ns indicates *p* > 0.05.

We next pooled all benthivorous (SB and LB) individuals as one group and compared allele frequencies SNP‐by‐SNP with data consisting of all pelagic (Pi and PL) individuals from Thingvallavatn. The analysis detected extremely differentiated SNPs (*p* < 1 × 10^−15^) across 37 scaffolds (Figure 5a). Moreover, similar to the observed contrast between dwarf and large benthivorous morphs, eight prominent outlier loci on six scaffolds exhibited a pattern suggesting putative inversions. These include large regions of 2.3 Mb (1429 SNPs with *p* < 1 × 10^−10^) and 2.7 Mb (896 SNPs) on scaffold 1; 2.3 Mb (787 SNPs) and 3.25 Mb (846 SNPs) on scaffold 3; 0.78 Mb on scaffold 8 (487 SNPs), 2.4 Mb on scaffold 9 (498 SNPs), 0.54 Mb on scaffold 14 (462 SNPs) and 0.76 Mb on scaffold 40 (259 SNPs) (Figure 5b–g). There is strong LD among alleles within the eight inversions with the top significant SNP, particularly on scaffold 1:16.3–18.6 Mb (*r* ^2^ mean ± SD: 0.39 ± 0.09), scaffold 1:19.5–22.2 Mb (*r* ^2^ mean ± SD: 0.31 ± 0.08), scaffold 3: 33.5–35.8 Mb (*r* ^2^ range: 0.2–0.6, mean ± SD: 0.35 ± 0.08), scaffold 3: 37.3–40.6 Mb (*r* ^2^ mean ± SD: 0.47 ± 0.07), scaffold 8 (*r* ^2^ mean ± SD: 0.42 ± 0.10), scaffold 9 (*r* ^2^ mean ± SD: 0.32 ± 0.06), scaffold 14 (*r* ^2^ range: 0.2–0.6, mean ± SD: 0.45 ± 0.08) and scaffold 40 (*r* ^2^ mean ± SD: 0.30 ± 0.05) (Figure 5b–g, Table S7). These regions harbour a total of 211 genes with 28 genes on scaffold 1:16.3–18.6 Mb, 42 on scaffold 1:19.5–22.2 Mb, 27 on scaffold 3: 33.5–35.8 Mb, 61 on scaffold 3: 37.3–40.6 Mb, 11 on scaffold 8, 23 on scaffold 9, 5 on scaffold 14 and 14 on scaffold 40 (Table S8). The most differentiated SNPs (*p* < 1 × 10^−15^) for each region and their effects in relation to the closest genes are summarised in Table S9.

We estimated nucleotide diversity per haplotype at each inversion locus using individuals homozygous for the benthic and pelagic inversion haplotypes (Tables S11 and S12; Figure 5h). The analysis revealed consistent and significantly lower nucleotide diversities (Wilcoxon test, *p* < 0.01) among benthic inversion haplotypes at five out of eight candidate inversions despite very similar genome‐wide nucleotide diversity in the two morph groups (Figure 5h).

The pattern of genotype distributions at the eight putative inversion loci was further explored using diagnostic markers (*p* < 1 × 10^−15^) among benthic versus pelagic morphs from Thingvallavatn (Figure S10). The genotype heatmap revealed distinct genotype patterns between the benthic and pelagic morphs. PCA conducted for each inversion region (including all SNPs) further supported this pattern, consistently clustering individuals by genotype class (Figure S11). Notably, the pattern of clear genotype‐based clustering held across all inversion regions except for the one located on scaffold 3: 37.3–40.6 Mb, where the separation between genotype classes was less distinct. Additionally, the analysis of genotype distributions showed that none of the putative inversions detected in Thingvallavatn is shared intact with the other lakes, suggesting that if these are true inversions, they do not have a wide geographic distribution. However, there were several examples of haplotype sharing for parts of the putative inversions for which the pattern was consistent across morphs and lake systems. Patterns of haplotype sharing were observed between Thingvallavatn and Mývatn morphs along three distinct regions (16,453,643‐16,910,602 bp; 17,026,128‐17,057,348 bp; 17,464,035‐17,653,321 bp) of the putative inversion on scaffold 1 (16.3–18.6 Mb). Additionally, similar patterns of differentiation were evident between Thingvallavatn and Sirdalsvatnet morphs along parts of the putative inversions 16.3–18.6 Mb (17,464,035‐17,653,321 bp) and 19.5–22.2 Mb (20,429,418‐20,718,588 bp) on scaffold 1, and within putative inversion 38.4–40.8 Mb (39,292,876‐39,384,683 bp) on scaffold 9. An exception to the rule that the small and large benthivorous morphs shared haplotypes at these putative inversions occurs at scaffold 8, where the large benthivorous morph shares haplotypes with the pelagic and piscivorous morphs (Figure S10).

### 3.5 Genetic Differentiation and Population Structure Between Morphs Across Lakes

The analysis of the putative inversion regions suggested the existence of some haplotype sharing between the same type of morphs across lakes. We therefore extended this analysis to the remaining part of the genome outside the putative inversions. Thingvallavatn was selected as the starting point for our analyses due to the clear signals of genetic differentiation among morphs in this lake.

We screened for the presence of shared patterns of differentiation between morphs from different lakes using the most differentiated genomic regions (*p* < 1 × 10^−15^) obtained in the dwarf/large benthivorous (Figure 4a) and benthic/pelagic morph contrasts (Figure 5a) from Thingvallavatn. Only two genomic regions outside the putative inversions showed strong differentiation between dwarf and large benthivorous morphs in Thingvallavatn, but no similar patterns were found across lakes (Figure S12).

On the other hand, a notable example of parallel genetic differentiation between lakes emerged in regions differentiating benthic and pelagic morphs, especially on scaffold 34 in the comparison between Thingvallavatn and Mývatn. The heatmap of allele frequency distributions across lakes (Figure S13) along with the corresponding predicted genotypes for individual fish (Figure S14a) showed that the small benthic morphs from Mývatn carried a haplotype for the region on scaffold 34: 18.36–18.45 Mb closely related to the one dominating among benthic morphs from Thingvallavatn. There was a moderate to strong LD (*r* ^2^ range: 0.0 to 0.88, mean ± s.d.: 0.26 ± 0.19) among genotypes within both lakes for this region (Figure S12b). This genomic region contains seven genes, *LRP10*, *MMP14*, *ACINU*, *AJUBA, CD244, FCRL5* and *RM52* with the currently known functions: LRP10 involved in lipid metabolism (Willnow 1999); heterochrony in MMP14 expression along with other genes was associated with terminal/benthic mouth phenotype in sucker fish (Searle et al. 2024); *ACINU* plays a role in splicing regulation as well as in other cellular pathways (Rodor et al. 2016); AJUBA may be involved in regulating cell growth and differentiation decisions during early development (Kanungo et al. 2000); *CD244* is an immunoregulatory receptor found on a variety of immune cells (Agresta et al. 2020); *FCRL5* is also involved in immune system regulation (Chorazy et al. 2021); and *RM52* plays a critical role in the maintenance of genome integrity under oxidative stress conditions (de Souza‐Pinto et al. 2009). A summary of the annotated genes and the most highly differentiated SNPs (*p* < 1 × 10^−15^) can be found in Tables S8 and S7, respectively.

To validate the presence of shared haplotypes, we carried out principal component analysis for individuals homozygous for the benthic (59 individuals) and pelagic (71 individuals) haplotypes from Thingvallavatn and Mývatn (Table S14). The analysis was based on a set of markers (5000 SNPs) located within the region 18.36–18.45 Mb of scaffold 34. The PCA explained a substantial proportion of variance (PC1: 33.3% and PC2: 22.8%) and illustrated distinct clustering of homozygous benthic and pelagic inversion haplotype samples from the two lakes (Figure S14c). Notably, PC2 further differentiated between benthic and pelagic individuals from Thingvallavatn and Mývatn, highlighting their genetic divergence despite observed haplotype sharing. To further explore this genetic structure, we constructed two neighbour‐joining phylogenetic trees of individuals being homozygous for benthic and pelagic haplotypes from the two lakes, both within and outside the shared region on scaffold 34 (Figure S14d,e). The clustering within the shared region (Figure S14d) was consistent with PCA results and followed the classification of morphs across lakes, whereas the results for the region outside the shared haplotype showed a clustering based on lake as for the rest of the genome (Figure S14e). The branch lengths separating the clusters of individuals with benthic haplotypes from different lakes or between pelagic haplotypes from different lakes were shorter than between haplotypes within lakes (Figure S14d). Pairwise *Fst* values among two benthic groups (0.11) and within two pelagic groups (0.21) were lower compared to those estimated across groups (Mývatn: *F*st = 0.81; Thingvallavatn: *F*st = 0.81). This region on scaffold 34 constitutes another possible example of a shared ancestral polymorphism contributing to morph differentiation in different lake systems.

## 4 Discussion

### 4.1 Establishing a Reference Genome Assembly for Arctic Charr

Here, we have established a high‐quality, scaffold‐level genome assembly for the Arctic charr and provide gene annotation based on short and long‐read RNA sequencing from multiple tissues including early developmental stages and cross‐species comparisons. This provides a major advance for omics studies of this Arctic fish as well as for its utilisation in aquaculture (Kess et al. 2021; Pappas et al. 2023).

### 4.2 Genetic Diversity in Arctic Charr

We found that the nucleotide diversity within populations was in the range 0.16%–0.22%, with higher values in Norwegian lakes (aged around 10,000 years) compared to Icelandic ones (aged around 2,300 years, Mývatn). Most of the sequence diversity observed among morphs must be much older than the postglacial lake colonisation within the last 10,000 years because it takes a much longer time to build up a nucleotide diversity exceeding 0.16%. Thus, the nucleotide diversity within populations reflects historical founder events and demographic processes following postglacial recolonisation of lake systems. It is possible, however, that secondary contact with divergent lineages has contributed to the level of genetic diversity, although the studied populations are not located near known contact zones in Atlantic Canada or Western Greenland and current mitochondrial data suggest that they fall within the core distribution of the Atlantic lineage (Brunner et al. 2001; Dallaire et al. 2025; Jacobsen et al. 2021; Salisbury et al. 2019).

### 4.3 The Genetic Basis of Morph Differentiation

The population structure among Arctic charr from two Icelandic and two Norwegian lakes based on whole genome resequencing is largely consistent with previous results on Arctic charr from the Northern hemisphere based on more sparse sets of genetic markers (reviewed by Salisbury and Ruzzante 2022). Firstly, populations clearly cluster by lakes rather than by morphs (Figure 2a). Our data are consistent with a scenario of early postglacial isolation of these Arctic charr populations in the four lakes as impassable waterfalls formed downstream of the lakes (Hindar et al. 1986; Kapralova et al. 2011). Secondly, we revealed three levels of genetic differentiation among sympatric morphs. In Lake Vangsvatnet (i) no genetic differentiation was found (Figure 3d), implying that the phenotypic differentiation between the small benthic and planktivorous morphs in this lake is due to plasticity. In Lake Thingvallavatn (Figure 3h) and Mývatn (Figure 3f), we find (ii) clear genetic differentiation, localised in specific regions of the genome that far exceed the genomic background (Figure S6c,d–i), suggesting incomplete reproductive isolation and gene flow that tends to homogenise allele frequencies at neutral loci. Finally, in Lake Sirdalsvatnet, we find (iii) strong genetic differentiation across the entire genome (Figure 3a), consistent with complete or near complete reproductive isolation.

Why does the degree of genetic differentiation among morphs within lakes vary so much? Important factors include the time that has passed since a lake was colonised and chance events when critical steps towards differentiation occurred. Lake Vangsvatnet, estimated to be around 10,000 years old based on dating from a neighbouring fjord (Romundset et al. 2010), was likely colonised approximately 7,000 years BP (Jonsson and Hindar 1982). In contrast, Lake Sirdalsvatnet is much older, likely been ice‐free since 15,000 years BP. However, due to its location above high waterfalls, the exact timing of Arctic charr colonisation remains uncertain. Another possibility is that genetically differentiated populations of charr colonised the same lake. However, the most important factor is likely ecological conditions within lakes (Woods et al. 2013). This is illustrated by the two Norwegian lakes showing drastically different population structures. In Vangsvatnet, we found no genetic differentiation between morphs (Figure 3c,d), whereas the corresponding morphs in Sirdalsvatnet showed strong, genome‐wide divergence (Figure 3a,b). The results are in line with differences in spawning habits (timing and area), where in Vangsvatnet (60 m deep) the two morphs partly co‐occur in the spawning areas and may interbreed. Jonsson and Hindar (1982) reported that some small benthic females were found together with planktivorous males in shallow waters during spawning. In contrast, in Sirdalsvatnet (165 m deep) the majority of benthic and pelagic morphs spawn separately (the pelagic morph spawns at a depth of 0–32 m in November, whereas small benthic charr spawn at 55–70 m depth throughout the year (Hesthagen et al. 1995), most of them in June–September, Table S1) limiting possible gene flow. Morphs in both lakes exhibit habitat segregation for significant portions of the year. However, during periods of food surplus, this segregation seems to diminish in Vangsvatnet (Hindar and Jonsson 1982). Consistent with genetic differentiation, morphological studies suggest that the two morphs in Sirdalsvatnet differ more from each other in terms of the number of gill rakers, body size at sexual maturity, growth and number of eggs per body size (Hesthagen et al. 1995) than what the two morphs in Vangsvatnet do (Hindar and Jonsson 1982). In one of the deepest lakes in Norway, Tinnsjøen, in addition to dwarf benthic and planktivorous morphs, there is also a piscivorous morph, and a 5 cm long, colourless charr at the deepest part of the lake (460 m) (Østbye et al. 2020), similar to the one found in the 288 m deep Gander Lake, Canada (Kess et al. 2021; O'Connell and Dempson 2002). This illustrates how variation in environmental conditions impacts sympatric differentiation in Arctic charr.

We noted clear genetic differentiation between morphs in Lake Mývatn (Iceland) implying that gene flow must be limited. Although the two morphs appear to overlap in timing and location of spawning (Table S1), specifically within the main spawning ground of the generalist morph, microhabitat preferences for sites of spawning and size‐assortative mate choice most likely limit inter‐morph mating. Mývatn was formed around 2300 years ago (Einarsson et al. 2004). Thus, the Arctic charr in Mývatn has a relatively recent colonisation history compared to Thingvallavatn and the two Norwegian lakes. The genome‐wide changes observed in the Mývatn Arctic charr may include adaptive responses to various factors such as physical features of the benthic littoral zone and the extreme fluctuations of potential prey populations in the soft bottom and pelagic habitats of the main lake basins.

Thingvallavatn formed ~10,000 years ago, first as a glacial lake and later, following large lava flows from the northern and eastern sides, as a spring‐fed lake (Saemundsson 1992). The lake was likely colonised by charr shortly thereafter. Our analysis of nucleotide diversity revealed reduced genetic diversity within the Thingvallavatn populations (θ = 0.16%) as compared to other lakes (θ = 0.17%–0.22%). While all postglacial lake populations may have experienced founder effects, the timing and colonisation history can differ across lakes, and these factors influence levels of nucleotide diversity. Geological events of the postglacial period, such as large lava flows, opening and collapsing of rifts connected to the lake and abrupt changes of water levels (Saemundsson 1992), could have impacted the genetic connectivity of morphs and led to reductions in population size and genetic drift resulting in loss of genetic diversity. However, despite the lower nucleotide diversity, Thingvallavatn showed the most extensive phenotypic diversity with four distinct morphs present. This is most likely explained by the rich variety of habitats within this lake. The pattern of genetic differentiation among the four morphs present in Thingvallavatn seen in the genome‐wide data (Figure 3h) confirmed previous results based on much smaller sets of genetic markers (Gíslason et al. 1999; Guðbrandsson et al. 2019; Kapralova et al. 2011). While genetic differentiation between the large benthic, small benthic and planktivorous morphs was clear, the genome‐wide data showed that most piscivorous individuals were closely related to the planktivorous morph, but some showed intermediate or closer genetic affinities towards the large benthic morph (Figure 3h), a pattern consistent with previous reports (Guðbrandsson et al. 2019; Magnusson and Ferguson 1987; Volpe and Ferguson 1996; Xiao et al. 2024). A potential scenario is that the piscivorous charr in this lake have emerged and continue to emerge from the planktivorous fish via ontogenetic dietary shift to piscivory (Snorrason et al. 1994), a transition that leads to increased size at maturity thus opening the possibility of hybridisation with the large benthic morph. However, we cannot exclude the possibility that large benthic males could be misclassified as piscivorous charr due to the similar head morphology (elongated lower jaw‐hook). Some of these fish were found to be running in October when piscivorous charr were sampled (Guðbrandsson et al. 2019).

### 4.4 Identification of 12 Putative Inversions in Arctic Charr From Thingvallavatn

A major advantage of the present study (whole genome sequencing and the use of a high‐quality genome assembly) compared to previous studies using limited sets of markers is that the genome‐wide patterns of genetic differentiation become apparent. This resulted in the detection of as many as 12 putative inversions contributing significantly to the differentiation among morphs in Thingvallavatn (Figures 4 and 5). It is an open question whether these are true inversions or large haplotype blocks present due to reduced recombination and natural selection. It is challenging to identify inversion breakpoints based on short‐read data since inversions are often flanked by repeats (Wellenreuther and Bernatchez 2018). Thus, further characterisation of these putative inversions will require long‐read sequencing of haplotypes. The results of this study together with a previous report of a 1.2 Mb candidate inversion segregating in a population of Arctic charr in Northern Canada (Hale et al. 2021) add Arctic charr to a long list of fish species where putative or confirmed inversions play a significant role for intraspecies genetic differentiation. These include, for instance, Atlantic salmon where a large number of inversions have been reported (Bertolotti et al. 2020; Lien et al. 2016; Lubieniecki et al. 2010; Stenløkk et al. 2022), Atlantic herring in which four confirmed inversions contribute to ecological adaptation related to water temperature (Fuentes‐Pardo et al. 2024; Han et al. 2020; Jamsandekar et al. 2024), Atlantic silverside with 13 putative inversions (Akopyan et al. 2022) and European sprat with six putative inversions related to adaptation of this marine fish to brackish water bodies (Pettersson et al. 2024). Another example is the identification of large chromosomal inversions in rainbow trout associated with multiple adaptive traits (Pearse et al. 2019) or geographical distribution (Hale et al. 2024).

Notably, several genes located within the putative inversions identified in our study were also highlighted in relation to morph differentiation in previous transcriptomic and population genetic studies. For example, within the inversion on scaffold 4 detected in the contrast between small/large benthivorous morphs, the *WEE1*, *GAS1* and *DEN5A* genes (Table S8) were previously identified as genetically differentiated based on transcriptome sequencing (Guðbrandsson et al. 2019). *WEE1* is a critical regulator of cell cycle progression and mitosis (Nurse and Thuriaux 1980). *GAS1* is associated with embryonic cranial skeleton development and palate formation (Seppala et al. 2007), while *DEN5A* is involved in the negative regulation of neurite outgrowth (Han et al. 2016).

Among the genes located within putative inversions in the benthic/pelagic morph comparison (Table S8), a previous transcriptome study identified SNPs in *COX11* and *LRRC1* on scaffold 3 (37.3–40.6 Mb) that showed strong genetic differentiation between morphs, consistent with our findings (Guðbrandsson et al. 2019). Furthermore, *AP2* located within the putative inversion scaffold 8 was found to be differentially expressed between benthic and limnetic charr (Guðbrandsson et al. 2019). *AP2* was also previously shown to be associated with craniofacial development in zebrafish (Knight et al. 2005), which highlights its potential role in the formation of morph‐specific craniofacial traits in charr. Additionally, *TIMP2* on scaffold 3: 37.3–40.6 Mb, a gene primarily involved in regulating peripheral extracellular matrix remodelling (Ferreira et al. 2023) showed a 3.8‐fold higher expression in the developing head in charr embryos of benthic morphs compared with pelagic morphs from Lake Thingvallavatn (Ahi et al. 2014).

We noted a clear trend of differences in the level of nucleotide diversity between haplotypes at the 12 putative inversions detected in Thingvallavatn. The nucleotide diversity was consistently higher among the small benthic haplotypes compared with the large benthic haplotypes (Figure 4f). Similarly, the pelagic haplotypes had consistently higher nucleotide diversity than the benthic haplotypes (Figure 5h). In both cases, there was no significant difference between morphs in genome‐wide nucleotide diversity, making it highly unlikely that differences in demographic history of the morphs explain the difference at the putative inversions. A reduced nucleotide diversity for one of the alleles at an inversion locus may reflect that this is the derived allele and/or that it has experienced a recent selective sweep.

### 4.5 Arctic Charr as a Model in Evolutionary Biology

An important question addressed in this study is the genetic basis for the characteristic sympatric differentiation in Arctic charr and to which extent genetic differentiation is based on ancestral polymorphisms shared among lake systems as well as genetic parallelism (i.e., how often the same or paralogous genes have contributed to adaptation). Firstly, it is clear that this species has an inherent ability to form phenotypically differentiated morphs even in the absence of genetic differentiation as noted for the Arctic charr in Vangsvatnet. This phenotypic plasticity is probably driven by the exploitation of different food resources benthic versus pelagic in particular (Andersson et al. 2005; Klemetsen et al. 2006; Skúlason et al. 1989). Secondly, genetic differentiation accumulates as some degree of reproductive isolation is established; for example, based on spawning location and/or spawning time (Adams et al. 2006). Genetic adaptation is likely to be highly polygenic given the large number of genomic regions showing high differentiation in the current and previous studies (Salisbury and Ruzzante 2022), compared with the genomic background. This can be expected, given that Arctic charr morphs show phenotypic differentiation in multiple diverse traits such as feeding morphology, growth patterns, feeding behaviour and spawning time (Jónsdóttir et al. 2024; Sandlund et al. 1992). However, neoteny has been suggested as an important mechanism of morph formation as it influences many of the traits involving allometric growth and life history (Eiríksson et al. 1999; Skúlason et al. 1989) and could in principle be driven by changes in one or few regulatory loci.

Our comparison across lakes and morphs revealed that none of the 12 putative inversions showing strong genetic differentiation among the four morphs in Thingvallavatn segregated intact, with the same putative breakpoints, in any of the other three lakes. Further, the majority of the signals of selection outside the putative inversions do not appear to be shared among lakes. Thus, genetic differentiation of benthic and pelagic morphs in different lake systems show only limited sharing of specific haplotypes associated with morph differentiation, for several possible reasons. The long time since the lakes were isolated, means independent evolution (no or limited gene flow between geographically isolated lakes), the lakes are quite different in topography and ecology and the phenotypes of the morphs differ considerably (lack of morphological parallelism). However, a notable exception is a 90 kb region on scaffold 34, which represents a shared region of differentiation between benthic and pelagic morphs from Thingvallavatn and Mývatn, highlighting potential common adaptations across the Icelandic lakes (Figure S14).

Additionally, we found clear indications of haplotype blocks of smaller segments within the putative inversion showing consistent morph associations among lakes from Iceland and Norway. One example is the putative inversion on scaffold 9 showing strong differentiation between small and large morphs in Thingvallavatn, where more than half of this genomic region replicates a similar pattern in Sirdalsvatnet (Figure S8). A possible scenario for the evolution of the putative inversions present in Lake Thingvallavatn is that they contain a combination of closely linked polymorphisms, some ancestral, that interact epistatically. This is a classical scenario for the adaptive evolution of inversions (Kirkpatrick and Barton 2006). Haplotype sharing among lakes may in some cases represent ancestral inversions, but these inversion haplotypes have evolved by recombination, which change the exact breakpoints. Inversions drastically reduce recombination but not completely. For instance, the inversion determining the satellite male morph in the ruff wader arose by a recombination event between an ancestral inversion and a wild‐type chromosome (Hill et al. 2023).

The results of the present study strongly suggest that whole genome resequencing of sympatric morphs from many lake systems in the Northern hemisphere presents a great opportunity to dissect the most important genetic factors that have contributed to the remarkable morph differentiation of Arctic charr across the Arctic region. Low‐pass sequencing of thousands of individuals is a cost‐effective approach to explore genotype–phenotype relationships and for determining accurate population‐specific allele frequencies (Enbody et al. 2023). This combined with limited long‐read sequencing for the characterisation of major structural variations, has the potential to establish the Arctic charr as a model for adaptive evolution.

## Author Contributions

K.G. and Z.O.J. led the development of the reference assembly. L.A. led the population genomics project. K.K. was responsible for the population genomics analysis. A.C. constructed the Tn5 libraries for whole‐genome sequencing. M.E.P. contributed to the population genomics analysis. K.H., L.L. and N.R. collected, stored and provided DNA from the Norwegian samples for low pass sequencing. Z.O.J., S.S.S., A.P. and H.X. sampled the Icelandic lakes with the exception of the large morph of Mývatn. Z.O.J., S.S.S. and A.P. dissected the L.B. voucher sample for transcriptome sequencing. H.X. performed DNA extractions from all Icelandic samples used for low pass sequencing. G.D. and H.G.L. prepared the Hi‐C libraries under the supervision of H.S. M.O.F., K.B., T.B., L.C., C.C., M.A.D., A.I., N.I., V.K., S.L., S.M., D.S., R.M.W., C.W., J.C., J.M.D.W. and A.D. contributed to the development of the genome assembly. K.K. and L.A. wrote the paper with input from other co‐authors. All authors approved the manuscript before submission.

## Disclosure

Data Accessibility: All custom code used for processing and analysis of the whole‐genome re‐sequencing data is available in https://github.com/LeifAnderssonLab/Arctic_charr_morphs. Whole genome re‐sequencing data in this study have been deposited in the Sequence Read Archive (SRA) under BioProject accession number PRJNA1175689. Metadata are also stored in the (SRA) under BioProject accession number PRJNA1175689. Raw genomic reads used for reference genome assembly and annotation, assembly sequences and reference annotations are available from the European Nucleotide Archive under Project PRJEB76174.

Benefit‐Sharing Statement: This research was conducted in collaboration with scientists from Sweden, Norway, Iceland and the United Kingdom, who contributed genetic samples and reference genome assembly and are all included as co‐authors. The findings have been shared with the contributing communities and disseminated to the broader scientific community through publications and presentations (see above). The study addresses a priority concern by enhancing genomic resources for Arctic charr, thereby supporting future research in ecology, evolution and aquaculture.

## Conflicts of Interest

The authors declare no conflicts of interest.

## Supporting information


**Figure S1:** Syntenic regions identified between assembly scaffolds and the Canadian Arctic charr high‐density linkage map. GBS sequences assigned to sex‐specific linkage groups described in Nugent et al. (2017) were mapped to the 40 chromosome‐level scaffolds in the assembly. Linkage groups for the male (A) and female (B) maps are shown in rows while scaffolds are shown in columns. The figures in each cell represent the total number of GBS sequences shared between the corresponding linkage group and scaffolds. Syntenic blocks supported by three or more GBS sequences are highlighted in black. Empty cells indicate a lack of shared sequences.
**Figure S2:** Dot plot comparisons of chromosome‐level scaffolds and other *Salvelinus* sp assemblies using D‐GENIES (v1.5.0). The 40 chromosome‐level scaffolds are shown as a target on the x‐axis and other *Salvelinus* sp assemblies are shown as a query on the y‐axis. Genomic alignments representative of putative syntenic regions between assemblies are shown are colour‐coded lines reflecting the level of nucleotide identity (yellow: < 25%, orange: 25%–50%, green: 50%–75% and dark green: > 75%). (a) Dot plot analysis against chromosome‐level scaffolds from a possible hybrid with the Northern Dolly Varden (*S. malma malma*, accession number GCA_002910315.2) and (b) dot plot analysis against chromosome‐level scaffolds from a selectively bred line of Arctic charr ( *Salvelinus alpinus* ; accession number GCA_045679555.1).
**Figure S3:** Genetic differentiation among Arctic charr morphs in Lake Mývatn. (a) Scores of individuals along PC1 and PC2, and (b) ancestry proportions of individuals assuming two clusters (K = 2), estimated based on LD pruned 0.69 million SNPs (MAF > 0.05). The pre‐assignment of morphs was based on sampling habitats, but this analysis shows that this resulted in many misclassifications. The samples were therefore reclassified to reflect the two distinct clusters detected here and these were used in the subsequent genetic analysis. Morph abbreviations: Large generalist (LG) and Small benthic (Krús, SB).
**Figure S4:** Distribution of average sequencing depth across individual Arctic charr samples (*n* = 283). A total of 22 individuals showed average sequencing depth below 1X.
**Figure S5:** The genome‐wide sequencing depth distribution across all samples and chromosomes. Average sequencing depth was computed in 500 kb non‐overlapping windows. Local spikes in sequencing depth (e.g., on scaffolds 2, 13, 17, 22, 26 and 33) suggest the presence of some collapsed duplications, but no evidence for whole‐chromosome duplications was detected.
**Figure S6:** Genomic differentiation between morphs of Arctic charr from four lakes. Genome scan based on estimated allele frequencies for individual SNPs. (a) Sirdalsvatnet, (b) Vangsvatnet, (c) Mývatn and (d–i) Thingvallavatn. The x‐axis represents the scaffolds, and the y‐axis represents the significance value (−log10(*P*)‐value) per SNP. Each dot corresponds to a single SNP. The horizontal red line indicates the significance threshold based on Bonferroni correction with the adjusted significance level for *α =* 10^−3^. Morph abbreviations for Sirdalsvatnet and Vangsvatnet: Dwarf benthic (DB) and Large pelagic (LP); for Mývatn, Large generalist (LG) and Small benthic (Krús, SB), and Thingvallavatn, Piscivorous (Pi), Planktivorous (PL), Large benthivorous (LB) and Small benthivorous (SB).
**Figure S7:** Admixture analysis of individuals from Lake Thingvallavatn. (a) Ancestry proportions generated in NGSAdmix based on LD pruned set of 0.70 million SNPs (MAF > 0.05). Morph abbreviations: Piscivorous (Pi), Planktivorous (PL), Large benthivorous (LB) and Small benthivorous (SB). (b) Log‐likelihoods for 1–10 clusters in the admixture analysis. K = 3 was determined to best represent the genetic structure in the lake, aligning with the three distinct groups observed in the PCA plot (Figure 3g). Increasing K beyond 3 resulted in only minor changes in admixture proportions, suggesting that three main genetic groups capture the primary population structure within this lake. (c) Pairwise Fst values illustrating genetic differentiation between the four morphs.
**Figure S8:** Predicted genotypes based on genotype likelihoods from diagnostic markers at four putative inversions distinguishing large and small benthic morphs in Thingvallavatn. Genotype distributions sorted by lake and morph are shown for the putative inversion regions on scaffolds 4, 5, 9 and 17. Morph abbreviations for Sirdalsvatnet and Vangsvatnet: DB and LP; for Mývatn, LG and Small benthic (Krús, SB), and Thingvallavatn, Pi, PL, LB and SB. Individuals are coloured according to their estimated genotype. The black brackets indicate a shared pattern of differentiation between morphs.
**Figure S9:** Genetic differentiation among Arctic charr large and small benthivorous morphs in Lake Thingvallavatn. (a–d) Principal component scores (PC1 vs. PC2) of individuals based on SNPs located within putative inversion regions. Individuals are coloured according to their inferred genotype class (homozygous minor, heterozygous and homozygous major) as shown in Figure S8. Point shapes indicate morph groups.
**Figure S10:** Predicted genotypes based on genotype likelihoods from diagnostic markers at six putative inversions distinguishing Arctic charr benthic (large and small benthivorous) and pelagic (piscivorous and planktivorous) morphs in Thingvallavatn. Genotype distributions sorted by lake and morph are shown for the putative inversion regions on scaffolds 1, 3, 8, 9, 14 and 40. Morph abbreviations for Sirdalsvatnet and Vangsvatnet: DB and LP; for Mývatn, LG and Small benthic (Krús, SB), and Thingvallavatn, Pi, PL, LB and SB. Individuals are coloured according to their estimated genotype. The black brackets indicate a shared pattern of differentiation between morphs.
**Figure S11:** Genetic differentiation among Arctic charr benthic (large and small benthivorous) and pelagic (piscivorous and planktivorous) morphs in Lake Thingvallavatn. (a–h) Principal component scores (PC1 vs. PC2) of individuals based on SNPs located within putative inversion regions. Individuals are coloured according to their inferred genotype class (homozygous minor, heterozygous and homozygous major) as shown in Figure S10. Point shapes indicate morph groups.
**Figure S12:** Allele frequency of diagnostic SNPs outside the putative inversions selected based on the small/large benthivorous contrast of Arctic charr morphs from Thingvallavatn. Each column title denotes a scaffold, with each value representing a diagnostic marker. The colours of the column annotation panel were used to highlight clusters of spatially adjacent SNPs along the genome within the maximum distance between SNPs of 50 kb (Only regions containing more than four SNPs were included to ensure sufficient density for the heatmap. Each of the presented regions ranges in size from 0.116 to 0.146 Mb. SNPs were tracked based on the most common allele in the Small benthivorous morph from Thingvallavatn. Allele frequencies (AF) of these alleles are indicated by the colour code, grey colour indicates missing data. Morph abbreviations for Sirdalsvatnet and Vangsvatnet: DB and LP; for Mývatn, LG and (Krús) and Thingvallavatn, Pi, PL, LB and SB.
**Figure S13:** Allele frequency of diagnostic SNPs outside the putative inversions selected based on the benthic/pelagic contrast of Arctic charr morphs from Lake Thingvallavatn. Each row title denotes a scaffold, with each value representing a diagnostic marker. The colours of the column annotation panel were used to highlight clusters of spatially adjacent SNPs along the genome within the maximum distance between SNPs of 50 kb. Only regions containing more than four SNPs were included to ensure sufficient density for the heatmap. Each of the presented regions ranges in size from 0.167 to 0.314 Mb. SNPs were tracked based on the most common allele in the benthic morph from Thingvallavatn. Allele frequencies (AF) of these alleles are indicated by the colour code, grey colour indicates missing data. Morph abbreviations as in Figure S6.
**Figure S14:** Genetic differentiation at a region on scaffold 34 among Arctic charr morphs in Mývatn and Thingvallavatn. (a) Predicted genotypes based on genotype likelihoods from diagnostic markers at this region sorted by lake and morph. Morph abbreviations as in Figure 6. Individuals are coloured according to their estimated genotype. Each value in the column represents a diagnostic marker. SNPs were tracked based on the most common allele in benthic morphs from Thingvallavatn. (b) Zoom‐in profile of the genome‐wide scan based on estimated allele frequencies for individual SNPs and linkage disequilibrium represented as Pearson *r* ^2^ among genotypes in the diagnostic region on scaffold 34 (18.36–18.45 Mb). *r* ^2^ ranged 0 to 0.94, and on average was 0.16 ± 0.13 for Mývatn; and *r* ^2^ range: 0–0.88 and on average it was 0.26 ± 0.19 for Thingvallavatn. (c) PCA plot showing individual clustering of samples homozygous for benthic or pelagic haplotype from Mývatn and Thingvallavatn within the diagnostic region (scaffold 34: 18.36–18.45 Mb). (d) Neighbour‐joining tree of samples homozygous for benthic or pelagic haplotype from Mývatn and Thingvallavatn lakes within the diagnostic region (scaffold 34: 18.36–18.45 Mb) and (e) outside the diagnostic region (scaffold 34: 0.01–18.3 Mb and 18.5–40.7 Mb).
**Table S1:** Sample metadata, geological context and history of the studied lakes where distinct Arctic charr morphs occur, including spawning times of the study populations. The Norwegian samples were collected from 1980 to 1984 and were the same as those used by Hindar et al. (1986) and they are maintained in a frozen tissue bank kept by L.L and N.R. at the Department of Zoology, Stockholm University. The samples from Thingvallavatn were collected in 2016–2018. The samples of LG‐charr from Mývatn were taken in 2014 from a fisheries survey by the Freshwater Research Institute, and the samples of SB (Krús) charr were collected in 2015.
**Table S2:** Genome assembly data summary.
**Table S3:** Association table matching the 40 chromosome‐level scaffolds from *Salvelinus alpinus* (fSalAlp1.1.hap1.cur.20231016) to chromosome‐level scaffolds from a possible hybrid between Arctic charr and the Northern Dolly Varden (*S. malma malma*, GCA_002910315.2) using D‐GENIES (v1.5.0). Each row lists a scaffold in the query assembly (GCA_002910315.2) and the best matching scaffold in the target assembly (fSalAlp1.1.hap1.cur.20231016) with strand orientation and alignment coordinates.
**Table S4:** Association table matching the 40 chromosome‐level scaffolds from *Salvelinus alpinus* (fSalAlp1.1.hap1.cur.20231016) to chromosome‐level scaffolds from a selectively bred line of Arctic charr ( *Salvelinus alpinus* ; GCA_045679555.1) using D‐GENIES (v1.5.0). Each row lists a scaffold in the query assembly (GCA_045679555.1) and its best matching scaffold in the target assembly (fSalAlp1.1.hap1.cur.20231016) with strand orientation and alignment coordinates.
**Table S5:** Nucleotide diversity (θ) assessed across the whole genome among Arctic charr morphs and populations. Morph abbreviations for Sirdalsvatnet and Vangsvatnet: DB and LP; for Mývatn, LG and (Krús), and Thingvallavatn, Pi, PL, LB and SB.
**Table S6:** Number of scaffolds and SNPs with at least one marker exceeding the Bonferroni‐corrected significance threshold (α = 10^−3^), and genome‐wide average Fst for each contrast between Arctic charr morphs across four lakes.
**Table S7:** Linkage disequilibrium (*r* ^2^) statistics for outlier SNPs (*p* < 1 × 10^−10^) with the top significant SNPs within every inversion region among Arctic charr morphs from Lake Thingvallavatn.
**Table S8:** Gene list for regions within 5 kb upstream and 5 kb downstream of putative inversions distinguishing morphs in Thingvallavatn, including Scaffold 34 (18.36–18.45 Mb), associated with haplotypes distinguishing benthic and pelagic morphs in Mývatn and Thingvallavatn.
**Table S9:** Summary of the most differentiated SNPs (*p* < 1 × 10^−15^) for each diagnostic region, including their nearest gene, and relative position to genes (e.g., missense, synonymous, upstream, downstream or intergenic) as determined by snpEff.
**Table S10:** Genotype distribution at four putative inversions showing genetic differentiation between the small and large benthivorous and large benthic morphs present in Thingvallavatn.
**Table S11:** Nucleotide diversity (θ) assessed across the whole genome and at putative inversion regions among Arctic charr morphs from Thingvallavatn homozygous for small and large benthivorous haplotype.
**Table S12:** Genotype distribution at eight putative inversions showing genetic differentiation between the benthic (B) and pelagic (P) morphs present in Thingvallavatn.
**Table S13:** Nucleotide diversity (θ) assessed across the whole genome and at putative inversion regions among Arctic charr morphs from Lake Thingvallavatn homozygous for benthic or pelagic haplotype.
**Table S14:** Genotype distribution at locus 18.36–18.45 Mb of scaffold 34 showing genetic differentiation between morphs homozygous for benthic and pelagic haplotype present in Thingvallavatn and Mývatn.
**Methods S1**. Extended Materials and methods section for the reference genome assembly and annotation.

## Data Availability

All custom code used for processing and analysis of the whole‐genome re‐sequencing data are available in https://github.com/LeifAnderssonLab/Arctic_charr_morphs. Whole‐genome re‐sequencing data in this study have been deposited in the Sequence Read Archive under BioProject accession number PRJNA1175689. Raw genomic reads used for reference genome assembly and annotation, assembly sequences and reference annotations are available from the European Nucleotide Archive under Project PRJEB76174.
