# Supplementary material for: Whole Genome Sequencing Reveals How Plasticity and Genetic Differentiation Underlie Sympatric Morphs of Arctic Charr
Source: Mol Ecol. 2025 Aug 26;34(19):e70085. doi: 10.1111/mec.70085 (PMC12456120; doi:10.1111/mec.70085)
Supplement: Supplementary file 1 — Figure S1: Syntenic regions identified between assembly scaffolds and the Canadian Arctic charr high‐density linkage map. GBS sequences assigned to sex‐specific linkage groups described in Nugent et al. (2017) were mapped to the 40 chromosome‐level scaffolds in the assembly. Linkage groups for the male (A) and female (B) maps are shown in rows while scaffolds are shown in columns. The figures in each cell represent the total number of GBS sequences shared between the corresponding linkage group and scaffolds. Syntenic blocks supported by three or more GBS sequences are highlighted in black. Empty cells indicate a lack of shared sequences. Figure S2: Dot plot comparisons of chromosome‐level scaffolds and other Salvelinus sp assemblies using D‐GENIES (v1.5.0). The 40 chromosome‐level scaffolds are shown as a target on the x‐axis and other Salvelinus sp assemblies are shown as a query on the y‐axis. Genomic alignments representative of putative syntenic regions between assemblies are shown are colour‐coded lines reflecting the level of nucleotide identity (yellow: < 25%, orange: 25%–50%, green: 50%–75% and dark green: > 75%). (a) Dot plot analysis against chromosome‐level scaffolds from a possible hybrid with the Northern Dolly Varden ( S. malma malma, accession number GCA_002910315.2) and (b) dot plot analysis against chromosome‐level scaffolds from a selectively bred line of Arctic charr ( Salvelinus alpinus ; accession number GCA_045679555.1). Figure S3: Genetic differentiation among Arctic charr morphs in Lake Mývatn. (a) Scores of individuals along PC1 and PC2, and (b) ancestry proportions of individuals assuming two clusters (K = 2), estimated based on LD pruned 0.69 million SNPs (MAF > 0.05). The pre‐assignment of morphs was based on sampling habitats, but this analysis shows that this resulted in many misclassifications. The samples were therefore reclassified to reflect the two distinct clusters detected here and these were used in the subsequent genetic analy [file MEC-34-e70085-s001.zip › Supplementary Information.pdf]

MOLECULAR ECOLOGY

Supplemental Information for:

Whole genome sequencing reveals how plasticity and genetic differentiation underlie sympatric morphs of Arctic charr

Khrystyna Kurta[1,2], Mariano Olivera Fedi[3], Kendall Baker[3], Tom Barker[3], Leah Catchpole[3], Claudio Ciofi[4], Arianna Cocco[1], Joanna Collins[5], Genevieve Diedericks[6], Maria Angela Diroma[4], Alex Durrant[3], Kjetil Hindar[7], Alessio Iannucci[4], Naomi Irish[3], Vanda Knitlhoffer[3], Linda Laikre[8], Henrique G. Leitão[6], Sacha Lucchini[3], Seanna McTaggart[3], Arnar Pálsson[9], Mats E. Pettersson[1], Nils Ryman[8], Sigurður S. Snorrason[9], Hannes Svardal[6], David Swarbreck[3], Robert M. Waterhouse[10,11], Christopher Watkins[3], Jonathan M D Wood[5], Han H. Xiao[9], Karim Gharbi[3], Zophonías O. Jónsson[9], Leif Andersson[1, 12]

Correspondence and requests for materials should be addressed to Leif Andersson, Karim Gharbi or Zophonias Jónsson

Supplementary Methods 1.

Extended Materials and methods section for the reference genome assembly and annotation

Sampling for reference genome assembly. A large benthivorous charr from Lake Thingvallavatn was chosen for genome and transcriptome sequencing as part of the Pilot Project of the European Reference Genome Atlas (ERGA) initiative (Mc Cartney et al., 2024). LB charr were herded using a dragnet on the LB spawning grounds in Ólafsdráttur (DMM 64°13.8983'N, 21°03.1683'W) during the spawning season in late August. A single individual of the heterogametic male sex was caught by hand to serve as the ERGA reference sample. The fish was brought alive to the laboratory, euthanised with phenoxyethanol, photographed and immediately dissected for sampling from various tissues, strictly following ERGA guidelines. Tissue samples were snap-frozen in liquid nitrogen and stored at -80°C and shipped to the Earlham Institute and the University of Antwerp for processing. Voucher samples of fin and muscle were kept for archiving at the Icelandic Museum of Natural History. Sampling was done with license from the Icelandic Directorate of Fisheries and with permission from the Thingvellir National Park authority. Additional samples used for short-read RNA sequencing were from developmental time-series of offspring of LB charr parents from the same site, described in Matlosz *et al.* (2022), ENA project PRJEB45551).

Nucleic acid extraction. High-molecular weight DNA extraction was performed on snap-frozen spleen tissue using the Circulomics Nanobind Tissue Big DNA Kit. The extraction method was based on the Dounce protocol described in Circulomics protocol EXT-DHH-001. A total of 30 mg of tissue was used as input and split into five extractions with 6 mg input each to reduce viscosity. The eluted DNA was left at room temperature overnight and periodically mixed 5 times with a wide-bore 200 µL tip the following day. RNA was extracted from seven tissue types from the same individual as used for the genome assembly. The Omega EZNA Total RNA Kit I (R6834-01) was used for the initial attempt from all tissues, and those that performed poorly (testis, muscle, and blood) were repeated using the EZNA Total RNA Kit II (R6934-01). For all solid tissues, 20-30 mg input was used per extraction. For blood, the entire frozen sample was thawed in an approximately equal volume of RNA-Solv buffer from the EZNA Total RNA Kit II. This mixture was then

frozen at -80°C, and 250 µL was later taken for extraction. A 5-min GenoGrinder cycle with a 5mm steel bead was used for disruption of all tissue types, though the speed settings varied: 1000 rpm for gill, spleen, liver, and brain, and 1250 rpm for blood, testis, and muscle. To ensure small RNAs were not excluded from the final sample, the precipitation step following homogenization was performed with 100% ethanol instead of the recommended 70%.

Illumina RNA sequencing. Stranded mRNA-seq libraries were constructed at the Earlham Institute using the NEBNext Ultra II RNA Library prep for Illumina kit (NEB#E7760L), NEBNext Poly(A) mRNA Magnetic Isolation Module (NEB#7490) and NEBNext Multiplex Oligos for Illumina (96 Unique Dual Index Primer Pairs) (E6440S) and sequenced at a concentration of 10µM. 1 µg of RNA was purified to extract mRNA with a Poly(A) mRNA Magnetic Isolation Module. Isolated mRNA was then fragmented for 12 min at 94°C, and converted to cDNA. NEBNext Adaptors were ligated to end-repaired, dA-tailed DNA. The ligated products were subjected to a bead-based purification using Beckman Coulter AMPure XP beads (A63882) to remove most un-ligated adaptors. Adaptor Ligated DNA was then enriched by receiving 10 cycles of PCR (30 s at 98°C, 10 cycles of: 10 s at 98°C, 75 s at 65°C 5 min at 65°C, final hold at 4°C). The size of the resulting libraries was determined using Agilent High Sensitivity DNA Kit from Agilent Technologies (5067-4626) and the concentration measured with a High Sensitivity Qubit assay from ThermoFisher (Q32854). The final libraries were pooled equimolarly and quantified by qPCR. The pool was diluted down to 0.5 nM using EB (10mM Tris pH8.0) in a volume of 18µl before spiking in 1% Illumina phiX Control v3. This was denatured by adding 4µl 0.2N NaOH and incubating at room temperature for 8 min, after which it was neutralised by adding 5µl 400mM tris pH 8.0. A master mix of DPX1, DPX2, and DPX3 from Illumina's Xp 2-lane kit was made and 63ul added to the denatured pool leaving 90µl at a concentration of 100pM. This was loaded onto a single lane of the NovaSeq SP flow cell (v1.5) using the NovaSeq Xp Flow Cell Dock before loading onto the NovaSeq 6000. The NovaSeq was run using NVCS v1.7.5 and RTA v3.4.4 and was set up to sequence 150bp PE reads. The data was demultiplexed and converted to fastq using bcl2fastq2**.**

Pacific Biosciences HiFi genome sequencing. Five spleen high-molecular weight extractions were combined to construct and sequenced two libraries at the Earlham Institute using the SMRTbell Express Template Prep Kit 2.0 (PacBio, P/N 100-983-900). In total, 23.4 µg was split into two aliquots and manually sheared with the Megaruptor 3 instrument (Diagenode, P/N B06010003) according to the Megaruptor 3 operations manual. Each aliquot underwent AMPure PB bead (PacBio, P/N 100-265-900) purification and concentration before undergoing library preparation using the SMRTbell Express Template Prep Kit 2.0 (PacBio, P/N 100-983-900). The HiFi libraries were prepared according to the HiFi protocol version 03 (PacBio, P/N 101-853-100) and the final libraries were size fractionated using the SageELF system (Sage Science, P/N ELF0001), 0.75% cassette (Sage Science, P/N ELD7510). The libraries were quantified by fluorescence (Invitrogen Qubit 3.0, P/N Q33216) and the size of the library fractions were estimated from a smear analysis performed on the FEMTO Pulse System (Agilent, P/N M5330AA). The libraries were sequenced on the Sequel IIe across eight Sequel II SMRT cells 8M. The parameters for sequencing per SMRT cell were: Adaptive loading default settings, 30-h movie, 2-h pre-extension time, 80-90pM on plate loading concentration. The loading calculations for sequencing were completed using the PacBio SMRTLink Binding Calculator 10.2. Sequencing primer v5 was annealed to the adapter sequence of the HiFi libraries. The libraries were bound to the sequencing polymerase with the Sequel II Binding Kit v2.2 (PacBio, P/N 102-089-000). Calculations for primer and polymerase binding ratios were kept at default values for the library type. Sequel II DNA internal control 1.0 was spiked into each library at the standard concentration prior to sequencing. The sequencing chemistry used was Sequel II Sequencing Plate 2.0 (PacBio, P/N 101-820-200) and the Instrument Control Software v 10.1.0.125432.

Pacific Biosciences Iso-Seq sequencing. The libraries were constructed and sequenced at the Earlham Institute starting from 231-348ng of total RNA per sample. Reverse transcription cDNA synthesis was performed using NEBNext Single Cell/Low Input cDNA Synthesis & Amplification Module (NEB, E6421). Each cDNA sample was amplified with barcoded primers for a total of 12 cycles. The barcoded cDNA samples were pooled equimolar into a 3-plex and 4-plex before SMRTbell library construction. Each library pool was prepared according to the guidelines laid out in the Iso-Seq protocol version 02 (PacBio, 101-763-800), using SMRTbell express template prep

kit 2.0 (PacBio, 102-088-900). The library pool was quantified using a Qubit Fluorometer 3.0 (Invitrogen) and sized using the Bioanalyzer HS DNA chip (Agilent Technologies, Inc.). Each Iso-Seq pool was sequenced on the Sequel IIe instrument with one Sequel II SMRT Cell 8M per pool. The parameters for sequencing were diffusion loading, 30-h movie, 2-h immobilisation time, 2-h pre-extension time, 60pM on plate loading concentration. The loading calculations for the Iso-Seq library pool using the PacBio SMRTlink Binding Calculator v 11.1.0.154383 and prepared for sequencing applicable to the library type. Sequencing primer v4 was annealed to the Iso-Seq library pool and complexed to the sequencing polymerase with the Sequel II binding kit V2.1 (PacBio, 101-843-000). Calculations for primer to template and polymerase to template binding ratios were kept at default values for the library type. Sequencing internal control complex 1.0 (PacBio, 101-717-600) was spiked into the final complex preparation at a standard concentration before sequencing for all preparations. The sequencing chemistry used was Sequel II Sequencing Plate 2.0 (PacBio, 101-820-200) and the Instrument Control Software v11.0.1.162970. We annotated protein-coding genes in the assembly using short-read RNA-Seq alignments and transcript assemblies from Pacific Biosciences Iso-Seq reads, including 467.8M RNA-Seq and 7.3M Iso-Seq reads from seven adult tissues and two development stages (Table S1), as well as cross-species alignment of protein sequences.

Dovetail Omni-C sequencing. A Hi-C library was prepared using Dovetail Genomics' Omni-C kit, following the manufacturer's protocol (v1.0) at the University of Antwerp. A liver tissue sample was manually homogenized with a micro pestle and needle and syringe before proceeding with the Hi-C library preparation steps. Library fragment size distribution and concentration were assessed using High Sensitivity D5000 ScreenTapes with a TapeStation 4150 (Agilent Technologies) and a Qubit Fluorometer (Thermo Fisher Scientific, Waltham, MA, USA), respectively. The Omni-C library was denatured and loaded for paired-end sequencing on an Illumina NovaSeq 6000 system using the S1 Reagent Kit v1.5 (300 cycles) at the University of Florence. We set a single index running mode to 6:151:151:0 bp cycles. Demultiplexing and conversion of sequencing data from bcl to fastq format were performed using bcl2fastq version 2.20 (Illumina).

Ploidy and genome size estimation. GenomeScope 2.0 (Ranallo-Benavidez et al., 2020) was used to generate a reference-free k-mer spectra profile and a smudgeplot to predict the ploidy of the species. First a meryl database was generated using a k-mer size of 21 (-k 21). The smudge plot was generated using kmers with a count > 200 and <3200.

Contig assembly. Hifiasm v0.18.5 (Cheng et al., 2021, 2022) was used to assemble the PacBio HiFi reads into a contiguous draft genome, using Omni-C reads to phase the genome into two phased genomes. A contamination screening was realized to the contigs of both assemblies using diamond v2.0.9 (Buchfink et al., 2021) blastx against the NCBI NR database (downloaded 29/03/2023) with the options --fast and --strand both to map against the positive and negative strands. The resulting hits were used as input for blobtools v1.0.1 (Laetsch & Blaxter, 2017) to generate a blobplot and a results table with the most likely phylogeny of each contig. Most of them were classified as Actinopterygii as expected for this species with some hits to other phylum. These contigs were manually blasted and the top hits were always from the Salmonidae family. Mito-Hi-Fi v2.2 (Uliano-Silva et al., 2023) was used to identify, extract and annotate the mitochondria from both assemblies. Only the mitochondria from the primary haplotype were kept for further analysis.

Scaffolding. YAhS v1.2 (Zhou et al., 2023) was used to scaffold the contigs using the Omni-C reads using a resolution of 1kb.

Manual curation. The final screened assembly was curated by the Genome Reference Informatics at the Wellcome Sanger Institute using the Omni-C data to generate a contact map and break/join scaffolds that had enough information to be associated as one DNA molecule.

Assembly k-mer spectra analysis. The quality and completeness of the assembly was assessed using merqury v1.3 (Rhie et al., 2020) using the meryl database created for the ploidy and genome size estimation step.

Repeat masking. Repeats were identified and masked using RepeatModeler v1.0.11 (Smit A.F.A. & Hubley R., 2015) and RepeatMasker v4.0.72 (Smit et al., 2015) via eirepeat v1.3.4 (https://github.com/EI-CoreBioinformatics/eirepeat).

Gene prediction. Gene models were annotated via the Robust and Extendable eukaryotic Annotation Toolkit (REAT, https://github.com/EI-CoreBioinformatics/reat) and Minos (https://github.com/EI-CoreBioinformatics/minos). The REAT workflow consists of three submodules: transcriptome, homology, and prediction. The transcriptome module utilised Illumina RNA-Seq data, mapping reads to the genome with HISAT2 v2.1.0 (Kim et al., 2019) followed by the identification of high-confidence splice junctions identified with Portcullis (Mapleson et al., 2018). The aligned reads were assembled for each tissue with StringTie2 v1.3.3 (Kovaka et al., 2019) and Scallop v0.10.2 (Shao & Kingsford, 2017). From the combined set of RNA-Seq assemblies a filtered set of non-redundant gene-models were derived using Mikado https://github.com/EI-CoreBioinformatics/mikado (Venturini et al., 2017). The REAT homology workflow was used to generate gene models based on alignment of protein sequences using Spaln2 (Iwata & Gotoh, 2012) and miniport (Li, 2023) from 10 related species previously annotated (Table S1) and a set of proteins downloaded from UniProt including all the proteins from the Actinopterygii class (taxid:7898). The prediction module generated evidence guided models based on transcriptome and protein alignments using AUGUSTUS v3.4.0 (Stanke & Morgenstern, 2005) (Stanke & Morgenstern, 2005) with three alternative configurations and weightings of evidence (see configs), Helixer (Stiehler et al., 2021) and EvidenceModeler v1.1.1 (Haas et al., 2008). Gene models from annotations of closely related organisms: *Salvelinus* spp., *Salvelinus namaycush*, *Salvelinus fontinalis* (GCF_002910315.2, GCF_016432855.1, GCF_029448725.1) were projected via Liftoff v1.5.1 (Shumate & Salzberg, 2020), and filtered via the multicompare script from ei-liftover pipeline (https://github.com/lucventurini/ei-liftover) ensuring only models with consistent gene structures between the original and transferred models were retained. The filtered Liftoff, REAT transcriptome, homology and prediction gene models were used in MINOS v1.9 (https://github.com/EI-CoreBioinformatics/minos) to generate a consolidated gene set with models selected based on evidence support and their intrinsic features (see config directory). Confidence and

biotype classification was determined for all gene models based on available evidence, such as homology support and expression (as defined in config_file). Transposable element gene classification was based on overlap with identified repeats (>40 bp repeat overlap).

Figure S1: Syntenic regions identified between assembly scaffolds and the Canadian Arctic Char high-density linkage map. GBS sequences assigned to sex-specific linkage groups described in (Nugent et al, 2017), were mapped to the 40 chromosome-level scaffolds in the assembly. Linkage groups for the male (A) and female (B) maps are shown in rows while scaffolds are shown in columns. The figures in each cell represent the total number of GBS sequences shared between the corresponding linkage group and scaffolds. Syntenic blocks supported by three or more GBS sequences are highlighted in black. Empty cells indicate a lack of shared sequences.

A

	1	2	3	4	5	6	7	8	9	10	11	12	13	14	15	16	17	18	19	20
AC-1+21m				1	1						93			1		1				
AC-2m															1					1
AC-3m	1			2					68											
AC-4p-m							1	1										1		
AC-4q-m	89		1																	
AC-5m						1			1		1			1						
AC-6m				1	90	1					1	1							1	
AC-7m																				
AC-8m						58														
AC-9m	2			2				1												
AC-10m		1		6			1													
AC-11m												13								
AC-12m										5		1								
AC-13/34m																				
AC-13m							72												1	
AC-14m								106												
AC-15m				104																
AC-16m			1				1											70		
AC-17m		1															54			
AC-18m			88	1																
AC-19m													1							
AC-20a-m		11																		
AC-20a/b-m		1																		
AC-20b-m		69						1												
AC-22m																				
AC-23m				1											60	1				
AC-24m									3											
AC-25m				1																
AC-26m														102						
AC-27m										55										
AC-28m							1													
AC-29m													60							
AC-30m																			29	
AC-31m	1																		1	
AC-32m						1														
AC-33m			1						1											96
AC-34m				1			2		1											
AC-35m					3															
AC-36m	1													1		69				
AC-37m													1							

	21	22	23	24	25	26	27	28	29	30	31	32	33	34	35	36	37	38	39	40
AC-1+21m	1			1	42						1							45		1
AC-2m	93												1							
AC-3m																			3	
AC-4p-m			3						1	39										
AC-4q-m	1													1						
AC-5m		88																		
AC-6m															1					
AC-7m									46											
AC-8m																				
AC-9m							72													
AC-10m								1				44								
AC-11m																				
AC-12m																				13
AC-13/34m													1							
AC-13m			2						1			1	1							
AC-14m			1						1											
AC-15m										1		5								
AC-16m																				
AC-17m				1				1												
AC-18m																				
AC-19m										1	79									
AC-20a-m																	24			
AC-20a/b-m																	1			
AC-20b-m							1										5			
AC-22m			44																	
AC-23m		2																		
AC-24m				1															33	
AC-25m																40				
AC-26m																				
AC-27m																				4
AC-28m								22												
AC-29m																				
AC-30m																				
AC-31m						63														
AC-32m				59																
AC-33m				1																
AC-34m					1						1		16							
AC-35m															57					
AC-36m										1										
AC-37m												1	1	35						

B

	1	2	3	4	5	6	7	8	9	10	11	12	13	14	15	16	17	18	19	20	21	22	23	24	25	26	27	28	29	30	31	32	33	34	35	36	37	38	39	40
AC-1+21f	1				1						**98**					1									**23**						1							**27**		
AC-2f		1																			**79**												1							
AC-3f	1	1		2					**39**					1																									**3**	
AC-4p-f																		1												**40**					1					
AC-4q-f	**156**		1		1						1															1				1										
AC-5f						1					1			1								**97**																		
AC-6f				2	**114**													1			1				1										**3**	1				
AC-7f																													**49**											
AC-8f						**66**																																		
AC-9f	2			2				1																			**76**													
AC-10f													1																			**24**								
AC-11f			1									**15**																												
AC-12f	1									**3**																								1						**22**
AC-13/34f																																	1							
AC-13f							**60**							1							1												2							
AC-14f								**123**															1						1											
AC-15f				**101**																1		1										**5**								
AC-16f			1															**86**																						
AC-17f						1											**50**																		1					
AC-18f			**87**																																	**3**	1			
AC-19f													1		1															1	**86**									
AC-20a-f		**10**			1								1																								**21**			
AC-20b-f		**140**						1																			1		1								**3**			
AC-22f																							**46**																	
AC-23f				1											**53**							2																		
AC-24f									**8**													1																	**26**	
AC-25f				1																																**39**				
AC-26f														**93**												1														
AC-27f										**82**																														**6**
AC-28f	1																							1				**49**												
AC-29f													**49**																											
AC-30f							1												**28**																					
AC-31f	1																									**71**			1											
AC-32f				1																				**89**																
AC-33f			1						1											**97**																				
AC-34f			1	1			1																										**19**							
AC-35f					**6**																														**42**					
AC-36f	1															**55**														1	1									
AC-37f																																	1	**58**						

Figure S2: Dot plot comparisons of chromosome-level scaffolds and other *Salvelinus sp* assemblies using D-GENIES (v1.5.0). The 40 chromosome-level scaffolds are shown as a target on the x-axis and other *Salvelinus sp* assemblies are shown as a query on the y-axis. Genomic alignments representative of putative syntenic regions between assemblies are shown are colour-coded lines reflecting the level of nucleotide identity (yellow: <25%, orange: 25–50%, green: 50–75%, and dark green: >75%). **(a)** dot plot analysis against chromosome-level scaffolds from a possible hybrid between Arctic charr with the Northern Dolly Varden (*S. malma malma*, accession number GCA_002910315.2) and **(b)** dot plot analysis against chromosome-level scaffolds from a selectively-bred line of Arctic charr (*Salvelinus alpinus*; accession number GCA_045679555.1)

a

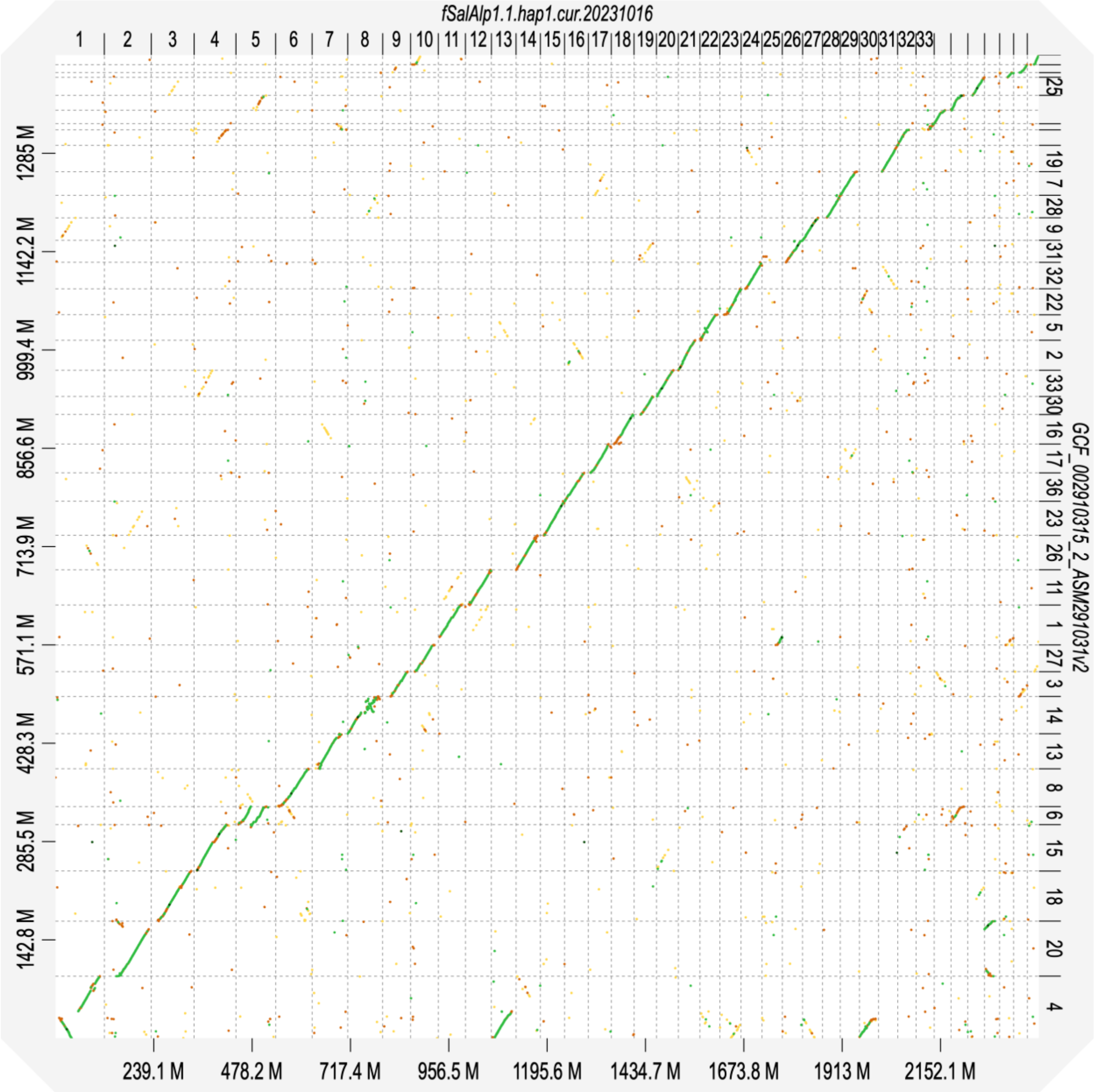

b

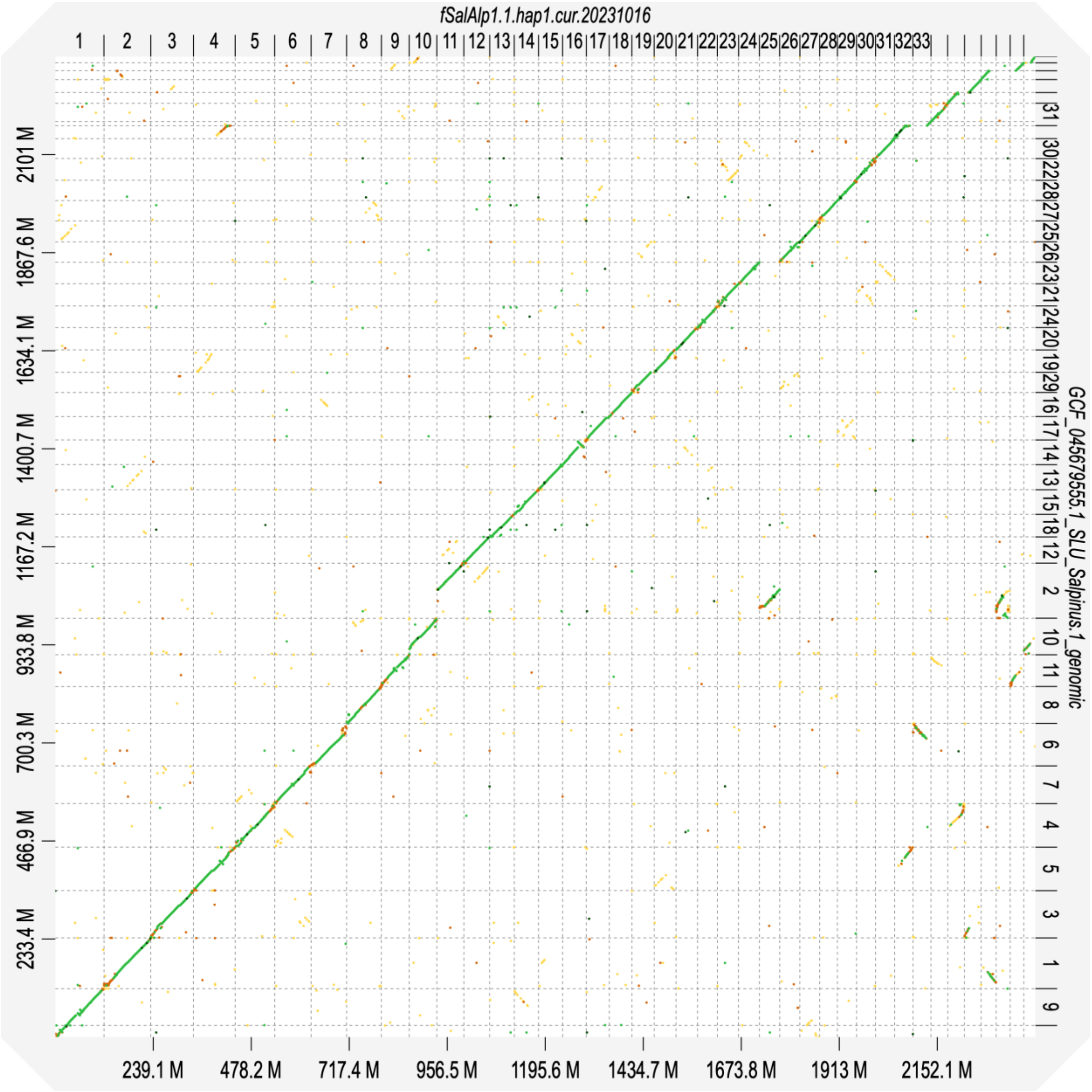

fSalAlp1.1.hap1.cur.20231016
1 2 3 4 5 6 7 8 9 10 11 12 13 14 15 16 17 18 19 20 21 22 23 24 25 26 27 28 29 30 31 32 33
GCF_045679555.1_SLU_Salpinus.1_genomic
31 30 22 28 27 25 26 23 21 24 20 19 29 16 17 14 13 15 18 12 2 10 11 8 6 7 4 5 3 1 9
2101 M
1867.6 M
1634.1 M
1400.7 M
1167.2 M
933.8 M
700.3 M
466.9 M
233.4 M
239.1 M
478.2 M
717.4 M
956.5 M
1195.6 M
1434.7 M
1673.8 M
1913 M
2152.1 M

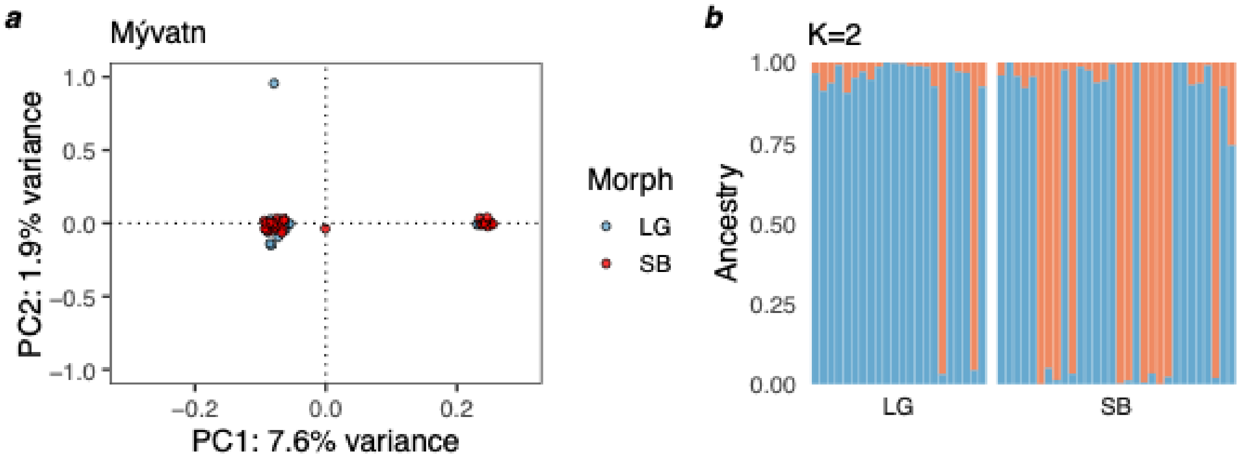

Figure S3: Genetic differentiation among Arctic charr morphs in Lake Mývatn. **(a)** Scores of individuals along PC1 and PC2, and **(b)** ancestry proportions of individuals assuming two clusters (K=2), estimated based on LD pruned 0.69 million SNPs (MAF > 0.05). The pre-assignment of morphs was based on sampling habitats but this analysis shows that this resulted in many misclassifications. To confirm the re-classification, we looked at the photographs of those individuals and verified the phenotypes to support the re-assignment The samples were therefore reclassified to reflect the two distinct clusters detected here and these were used in the subsequent genetic analysis. Morph abbreviations: Large generalist (LG) and Small benthic (Krús, SB).

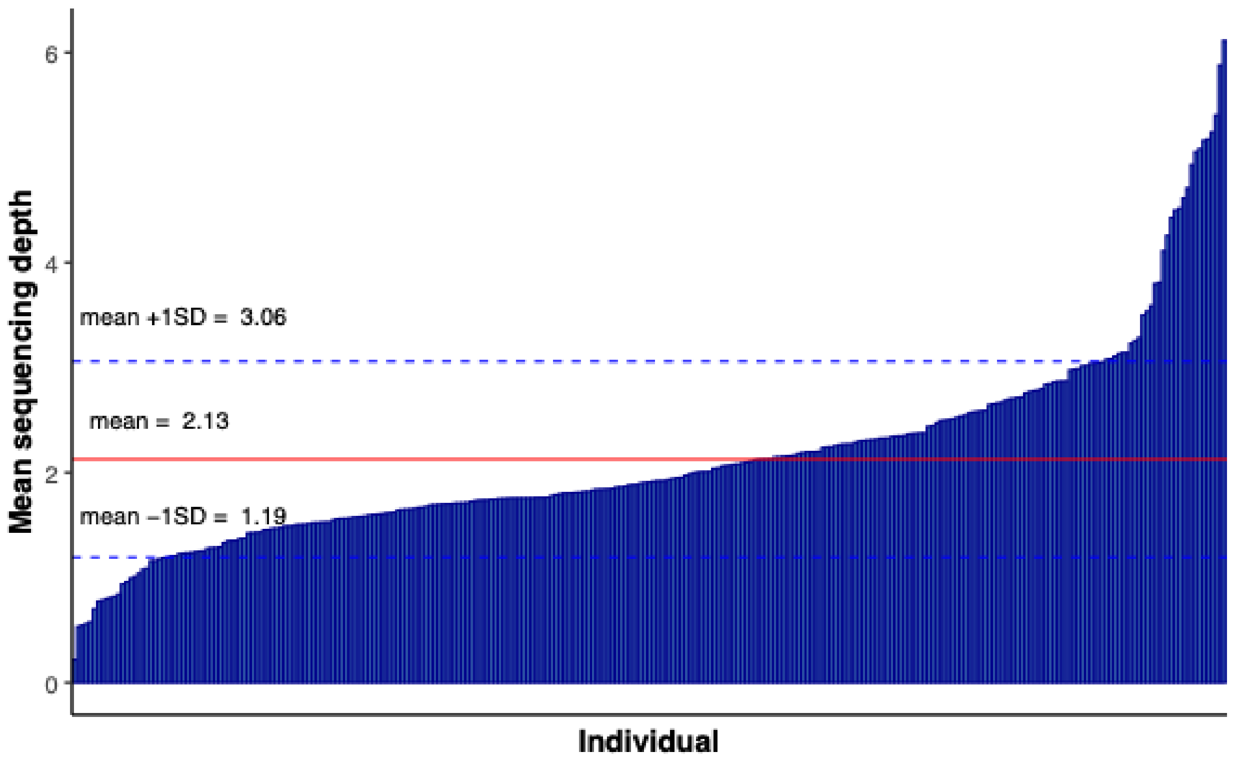

Figure S4: Distribution of average sequencing depth across individual Arctic charr samples (n = 283). A total of 22 individuals showed average sequencing depth below 1X.

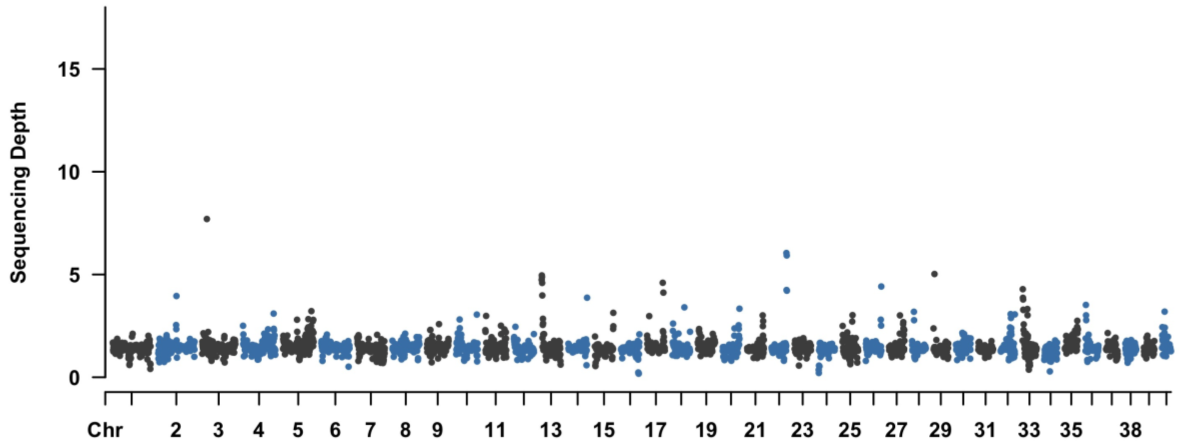

Figure S5: The genome-wide sequencing depth distribution across all samples and chromosomes. Average sequencing depth was computed in 500 kb non-overlapping windows. Local spikes in sequencing depth (e.g. on scaffolds 2, 13, 17, 22, 26, 33) suggest the presence of some collapsed duplications, but no evidence for whole-chromosome duplications was detected.

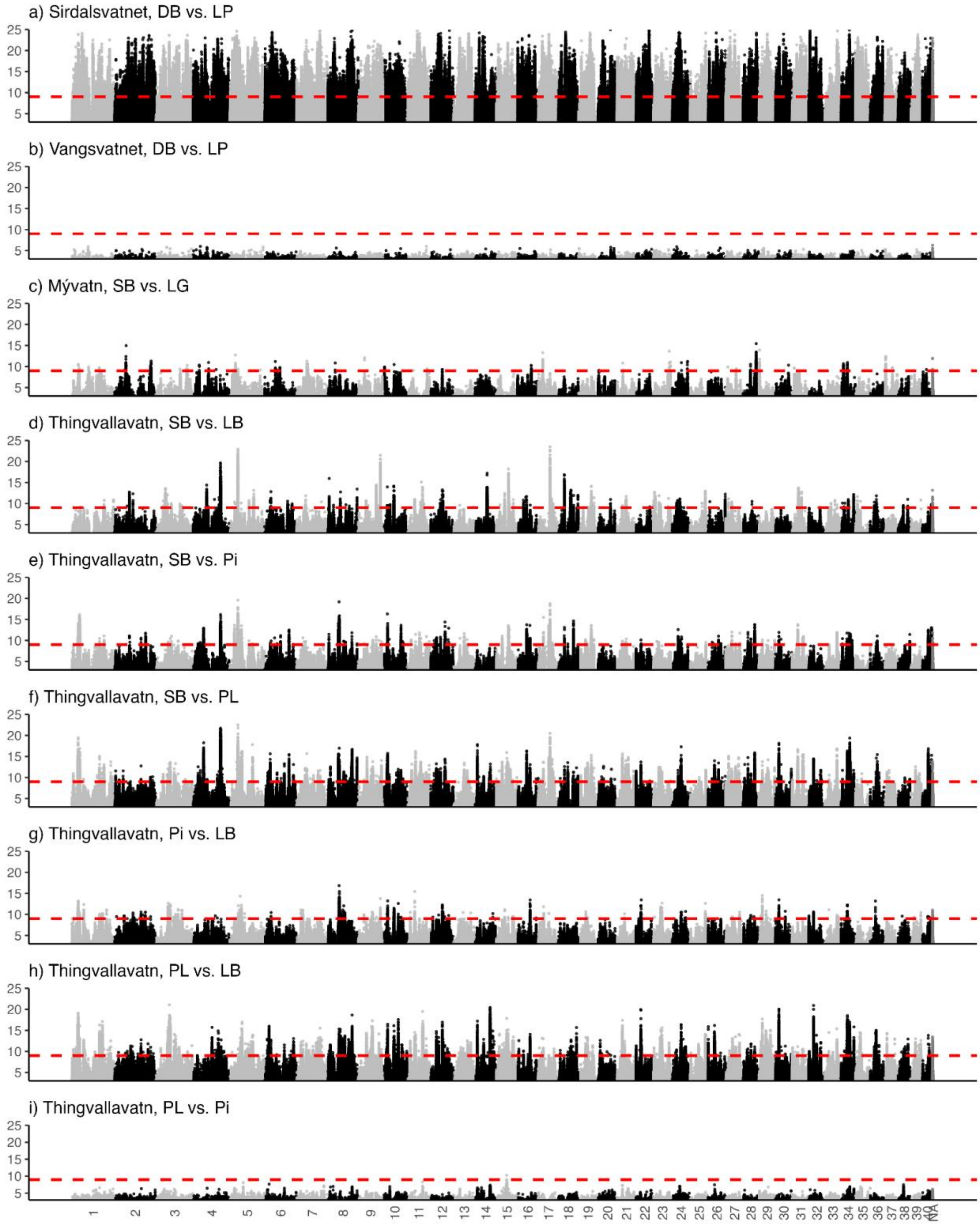

Figure S6: Genomic differentiation between morphs of Arctic charr from four lakes. Genome scan based on estimated allele frequencies for individual SNPs. (**a)** Sirdalsvatnet, (**b)** Vangsvatnet, (**c)** Mývatn, and (**d–i)** Thingvallavatn. The x-axis represents the scaffolds, and the y-axis represents

the significance value (-log10(*P*-value) per SNP. Each dot corresponds to a single SNP. The horizontal red line indicates the significance threshold based on Bonferroni correction with the adjusted significance level for $\alpha = 10^{-3}$. Morph abbreviations for Sirdalsvatnet and Vangsvatnet: Dwarf benthic (DB) and Large pelagic (LP); for Mývatn, Large generalist (LG) and Small benthic (Krús, SB), and Thingvallavatn, Piscivorous (Pi), Planktivorous (PL), Large benthivorous (LB) and Small benthivorous (SB).

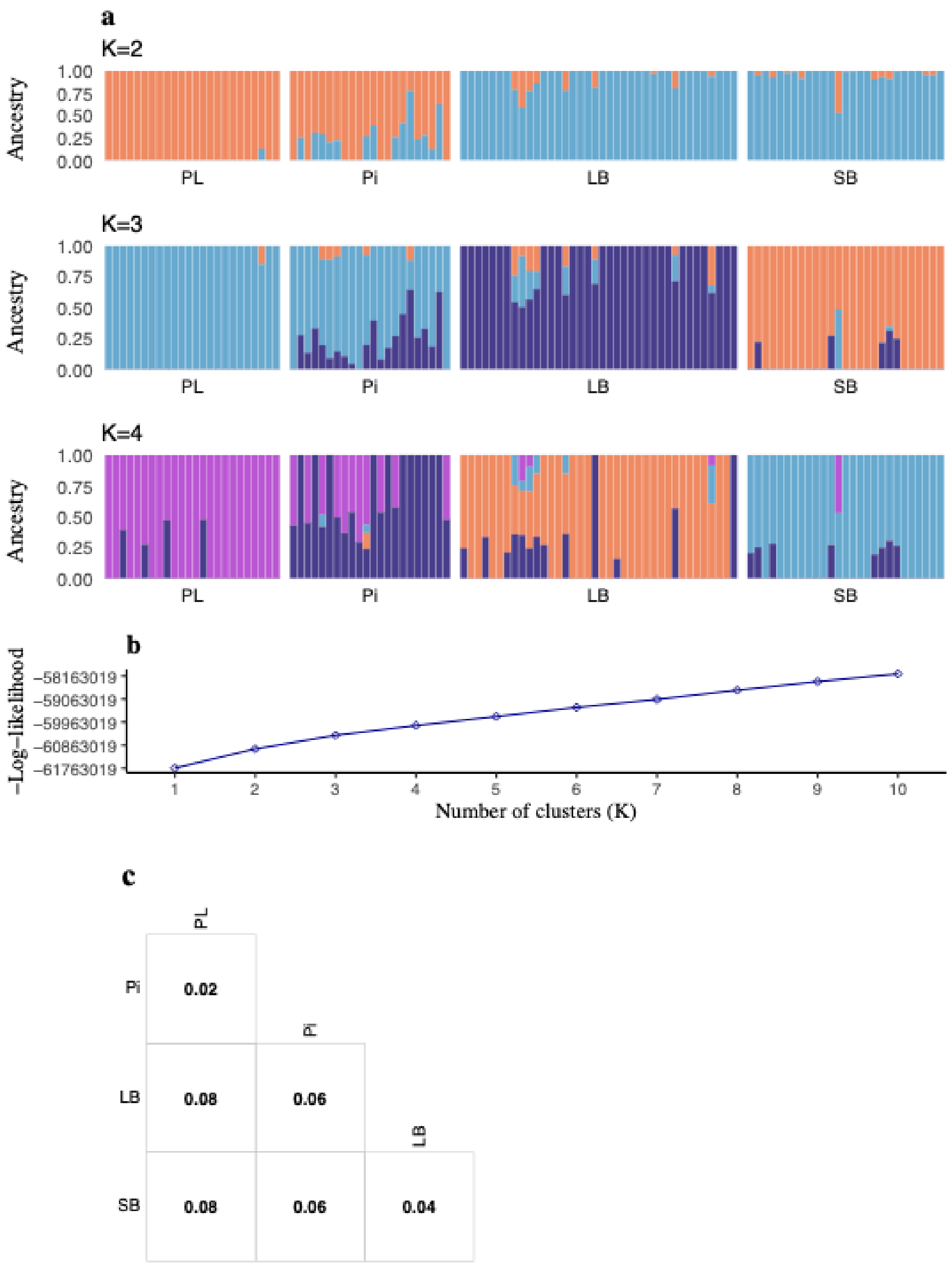

Figure S7: Admixture analysis of individuals from Lake Thingvallavatn. (a) Ancestry proportions generated in NGSAdmix based on LD pruned set of 0.70 million SNPs (MAF > 0.05). Morph abbreviations: Piscivorous (Pi), Planktivorous (PL), Large benthivorous (LB), and Small benthivorous (SB). (**b)** Log-likelihoods for 1–10 clusters in the admixture analysis. K = 3 was determined to best represent the genetic structure in the lake, aligning with the three distinct groups observed in the PCA plot (Figure 3g**)**. Increasing K beyond 3 resulted in only minor changes in admixture proportions, suggesting that three main genetic groups capture the primary population structure within this lake. (**c)** Pairwise Fst values illustrating genetic differentiation between the four morphs.

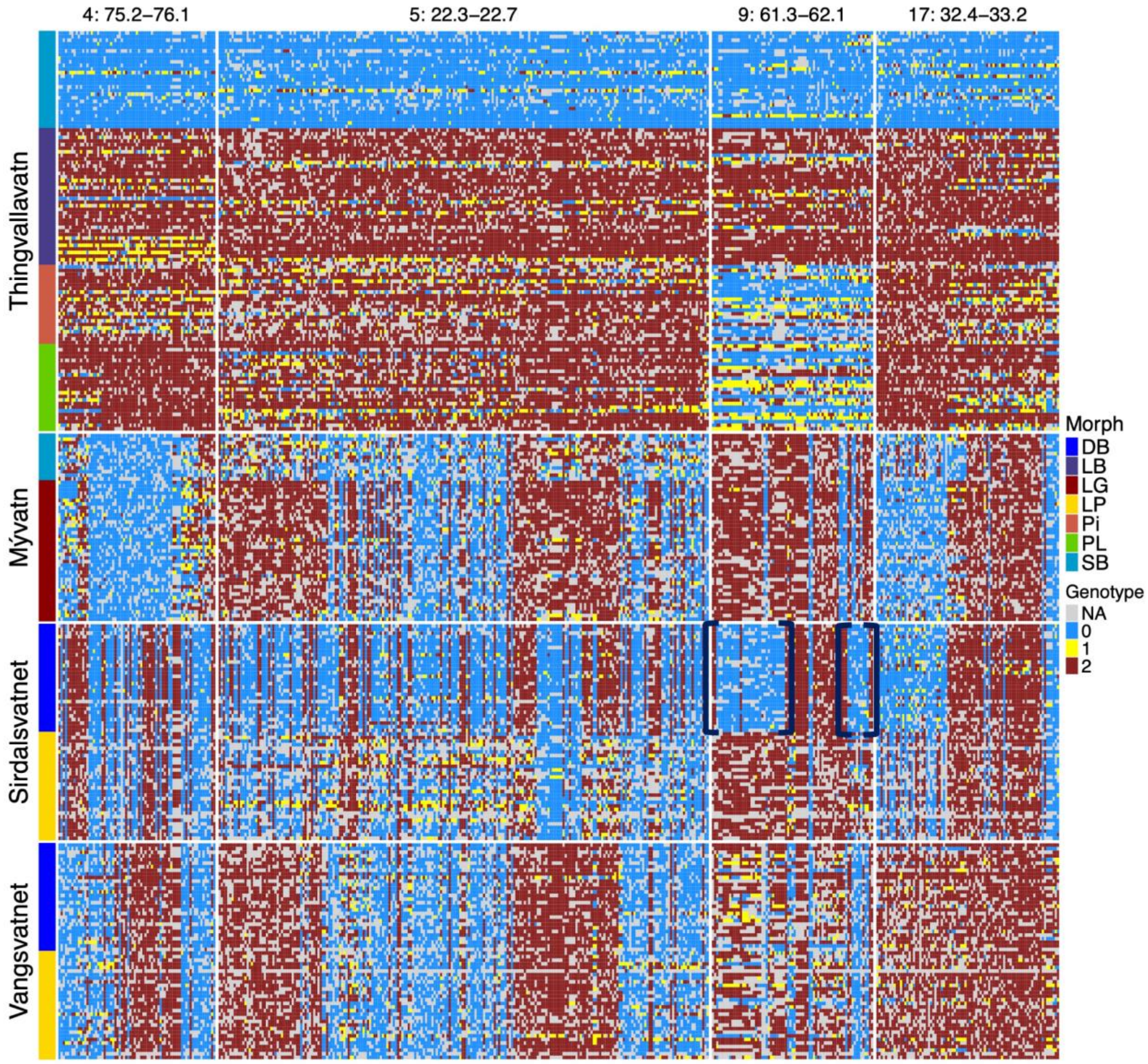

Figure S8: Predicted genotypes based on genotype likelihoods from diagnostic markers at four putative inversions distinguishing large and small benthivorous morphs in Thingvallavatn. Genotype distributions sorted by lake and morph are shown for the putative inversion regions on scaffolds 4, 5, 9, and 17. Morph abbreviations for Sirdalsvatnet and Vangsvatnet: Dwarf benthic (DB) and Large pelagic (LP); for Mývatn, Large generalist (LG) and Small benthic (Krús, SB), and Thingvallavatn, Piscivorous (Pi), Planktivorous (PL), Large benthivorous (LB) and Small benthivorous (SB). Individuals are colored according to their estimated genotype. The black brackets indicate a shared pattern of differentiation between morphs.

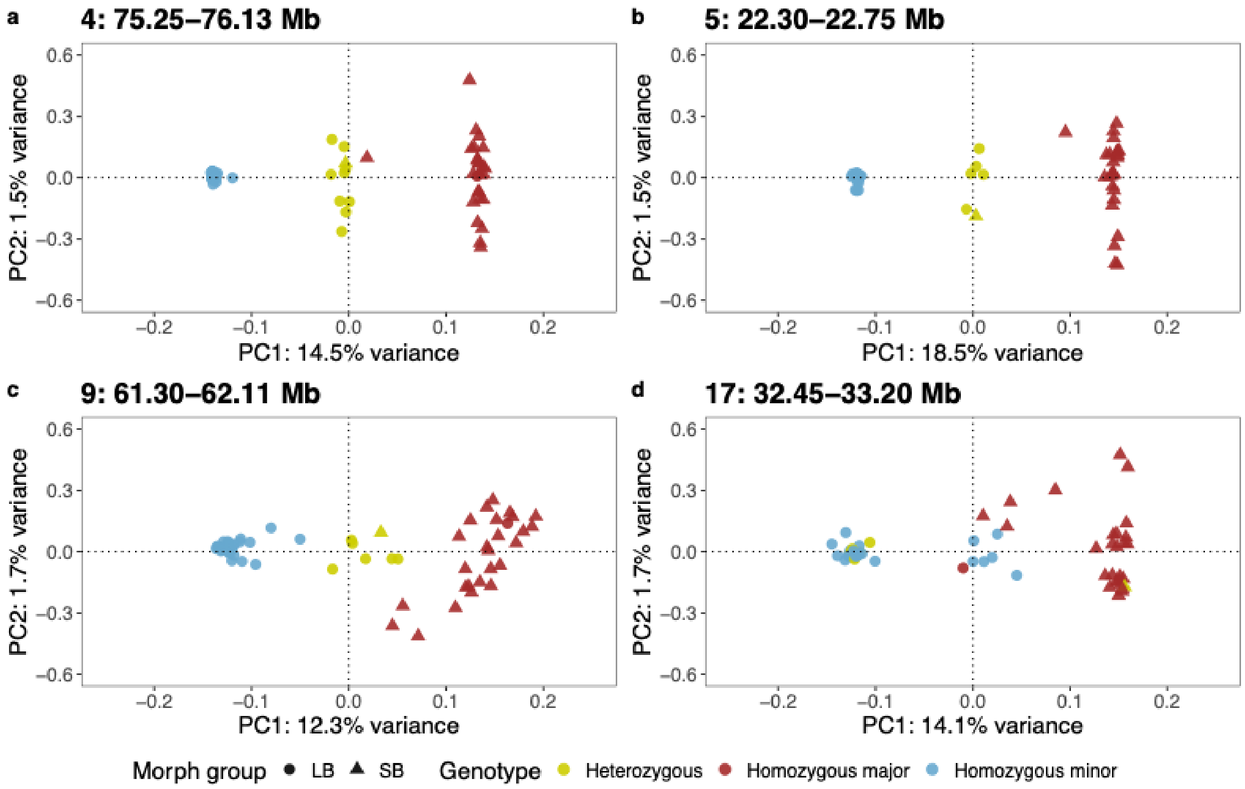

Figure S9: Genetic differentiation among Arctic charr large and small benthivorous morphs in Lake Thingvallavatn. **(a-d)** Principal component scores (PC1 vs. PC2) of individuals based on SNPs located within putative inversion regions. Individuals are colored according to their inferred genotype class (homozygous minor, heterozygous, homozygous major) as shown in Figure S9. Point shapes indicate morph groups.

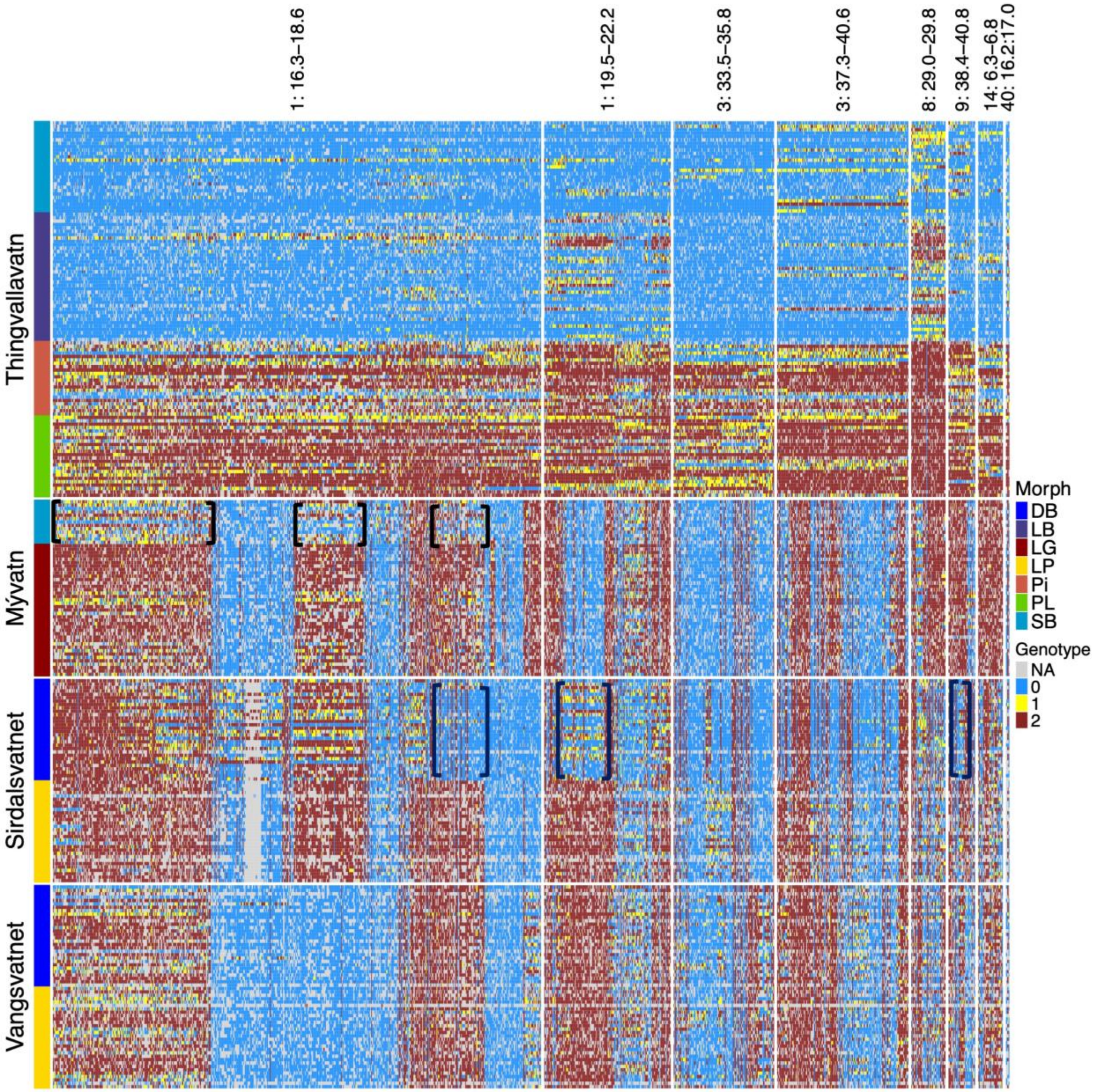

Figure S10: Predicted genotypes based on genotype likelihoods from diagnostic markers at six putative inversions distinguishing Arctic charr benthic (large and small benthivorous) and pelagic (piscivorous and planktivorous) morphs in Thingvallavatn. Genotype distributions sorted by lake and morph are shown for the putative inversion regions on scaffolds 1, 3, 8, 9, 14, and 40. Morph abbreviations for Sirdalsvatnet and Vangsvatnet: Dwarf benthic (DB) and Large pelagic (LP); for Mývatn, Large generalist (LG) and Small benthic (Krús, SB), and Thingvallavatn, Piscivorous (Pi), Planktivorous (PL), Large benthivorous (LB) and Small benthivorous (SB). Individuals are colored according to their estimated genotype. The black brackets indicate a shared pattern of differentiation between morphs.

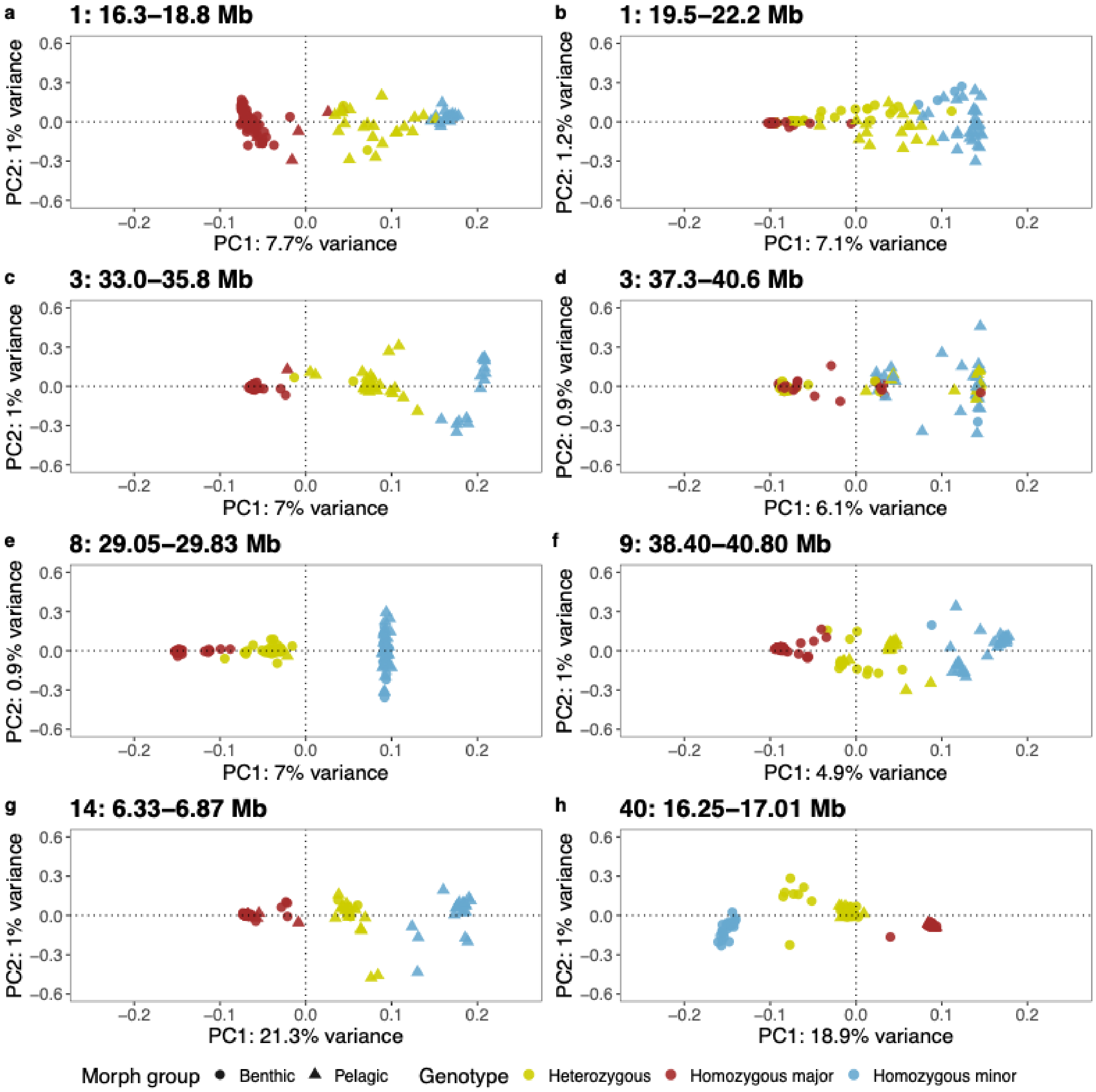

Figure S11: Genetic differentiation among Arctic charr benthic (large and small benthivorous) and pelagic (piscivorous and planktivorous) morphs in Lake Thingvallavatn. (a–h) Principal component scores (PC1 vs. PC2) of individuals based on SNPs located within putative inversion regions. Individuals are colored according to their inferred genotype class (homozygous minor, heterozygous, homozygous major) as shown in Figure S9. Point shapes indicate morph groups.

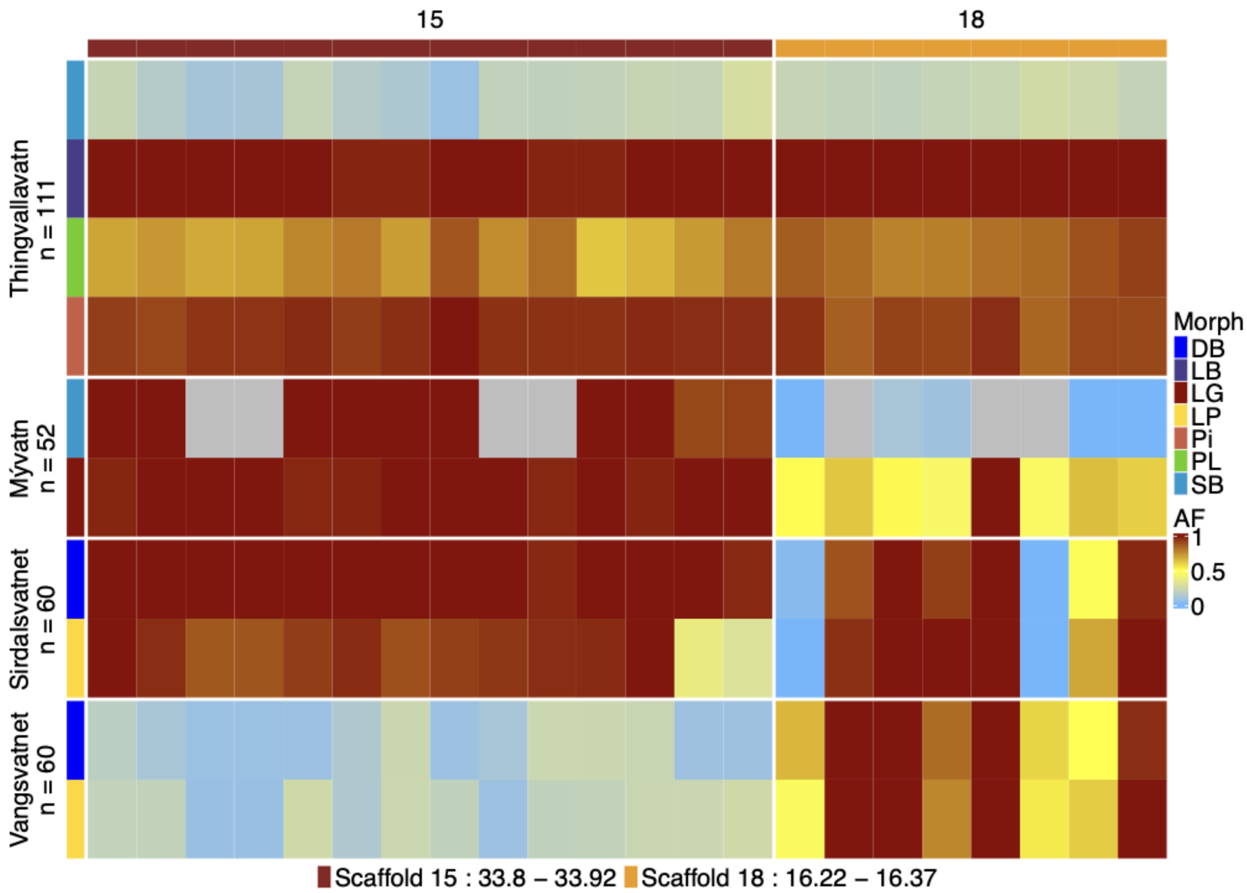

Figure S12: Allele frequency of diagnostic SNPs outside the putative inversions selected based on the small/large benthivorous contrast of Arctic charr morphs from Thingvallavatn. Each column title denotes a scaffold, with each value representing a diagnostic marker. The colors of the column annotation panel were used to highlight clusters of spatially adjacent SNPs along the genome within the maximum distance between SNPs of 50 kilobases (kb). Only regions containing more than four SNPs were included to ensure sufficient density for the heatmap. Each of the presented regions ranges in size from 0.116 to 0.146 Mb. SNPs were tracked based on the most common allele in the Small benthivorous morph from Thingvallavatn. Allele frequencies (AF) of these alleles are indicated by the color code, grey color indicates missing data. Morph abbreviations for Sirdalsvatnet and Vangsvatnet: Dwarf benthic (DB) and Large pelagic (LP); for Mývatn, Large generalist (LG) and Small benthic (Krús, SB), and Thingvallavatn, Piscivorous (Pi), Planktivorous (PL), Large benthivorous (LB) and Small benthivorous (SB).

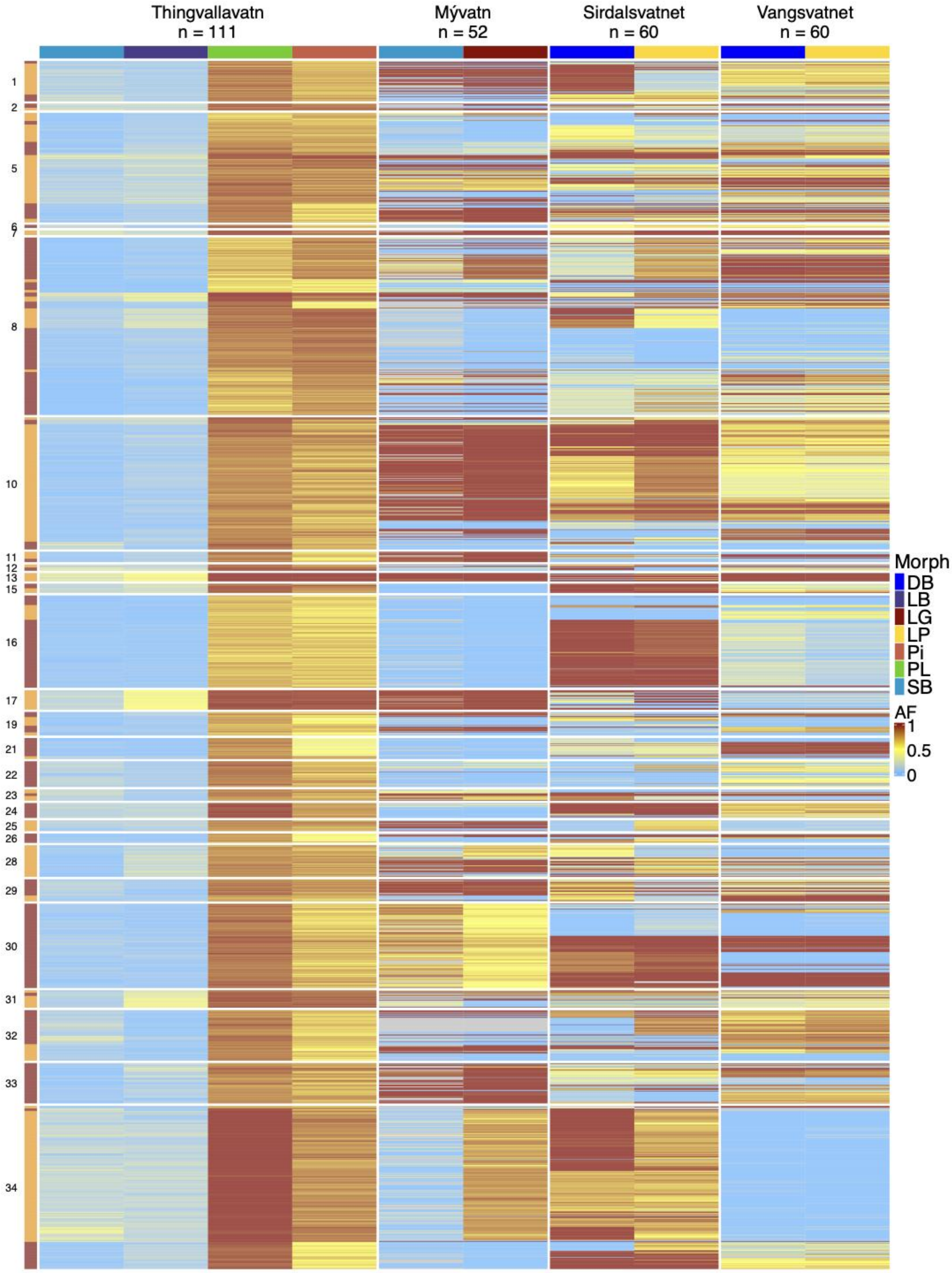

Thingvallavatn
n = 111
Mývatn
n = 52
Sirdalsvatnet
n = 60
Vangsvatnet
n = 60
1
2
5
6
7
8
10
11
12
13
15
16
17
19
21
22
23
24
25
26
28
29
30
31
32
33
34
Morph
DB
LB
LG
LP
Pi
PL
SB
AF
1
0.5
0

Figure S13: Allele frequency of diagnostic SNPs outside the putative inversions selected based on the benthic/pelagic contrast of Arctic charr morphs from Lake Thingvallavatn. Each row title denotes a scaffold, with each value representing a diagnostic marker. The colors of the column annotation panel were used to highlight clusters of spatially adjacent SNPs along the genome within the maximum distance between SNPs of 50 kilobases (kb). Only regions containing more than four SNPs were included to ensure sufficient density for the heatmap. Each of the presented regions ranges in size from 0.167 to 0.314 Mb. SNPs were tracked based on the most common allele in the benthic morph from Thingvallavatn. Allele frequencies (AF) of these alleles are indicated by the color code, grey color indicates missing data. Morph abbreviations as in Figure 6.

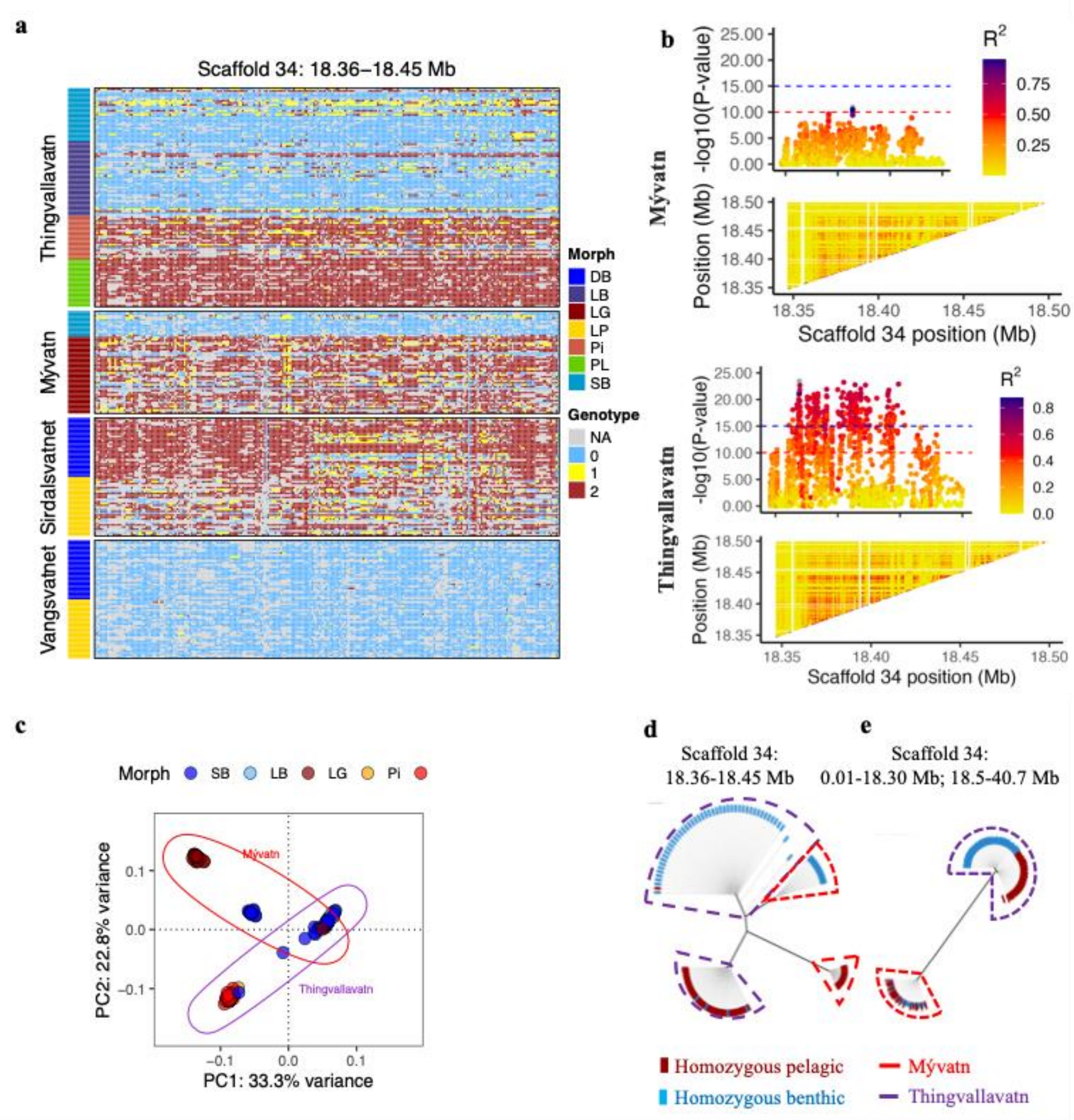

Figure S14: Genetic differentiation at a region on scaffold 34 among Arctic charr morphs in Mývatn and Thingvallavatn. (a) Predicted genotypes based on genotype likelihoods from diagnostic markers at this region sorted by lake and morph. Morph abbreviations as in Figure 6. Individuals are colored according to their estimated genotype. Each value in the column represents a diagnostic marker. SNPs were tracked based on the most common allele in benthic morphs from Thingvallavatn. **(b)** Zoom-in profile of the genome-wide scan based on estimated allele frequencies for individual SNPs and linkage disequilibrium represented as Pearson R^2 among genotypes in the diagnostic region on scaffold 34 (18.36-18.45 Mb). R^2 ranged 0 to 0.94, and on average was 0.16 ± 0.13 for Mývatn; and R^2 range: 0-0.88, and on average it was 0.26 ± 0.19 for Thingvallavatn. **(c)** PCA plot showing individual clustering of samples homozygous for benthic

or pelagic haplotype from Mývatn and Thingvallavatn within the diagnostic region (scaffold 34: 18.36-18.45 Mb). **(d)** Neighbor-joining tree of samples homozygous for benthic or pelagic haplotype from Mývatn and Thingvallavatn lakes within the diagnostic region (scaffold 34: 18.36-18.45 Mb) and **(e)** outside the diagnostic region (scaffold 34: 0.01-18.3 Mb and 18.5 - 40.7 Mb).

Table S1 Sample metadata, geological context, and history of the studied lakes where distinct Arctic charr morphs occur, including spawning times of the study populations. The Norwegian samples were collected from 1980 to 1984 and were the same as those used by Hindar et al. (1986) and they are maintained in a frozen tissue bank kept by L.L and N.R. at the Department of Zoology, Stockholm University. The samples from Thingvallavatn were collected in 2016-2018. The samples of LG-charr from Mývatn were taken in 2014 from a fisheries survey by the Freshwater Research Institute, and the samples of SB (Krús) charr were collected in 2015.

Country	Location	Age in years, post-glacial period	Max depth, m	Morph	Abbreviated name	Lake zone	Sample size	Sample reference	Spawning
Norway	Vangsvatnet	~10,000	60	Large pelagic	LP	Pelagic	30	(Hindar et al. 1986)	late October-early December, shallow water (Jonsson & Hindar, 1982)
Norway	Vangsvatnet	-	-	Dwarf benthic	DB	Benthic	30	(Hindar et al.1986)	late October-early December, shallow water (Jonsson & Hindar, 1982)
Norway	Sirdalsvatnet	~10,000	165	Large pelagic	LP	Pelagic	30	(Hindar et al.1986)	late October -November, 0-32 m (Jonsson & Jonsson, 2001)
Norway	Sirdalsvatnet	-	-	Dwarf benthic	DB	Benthic	30	(Hindar et al., 1986)	throughout the year, peaks June-September, 55-70 m (Jonsson & Jonsson, 2001)
Iceland	Thingvallavatn	~10 000 (Saemundsson, 1992)	114	Large benthivorous	LB	Benthic	38	Not published	Peak in late July - early August. Stony littoral zone near cold springs (Skúlason et al., 1989)
Iceland	Thingvallavatn	-	-	Small benthivorous	SB	Benthic	27	Not published	Variable, early September – November. Stony littoral zone. (Skúlason et al., 1989)
Iceland	Thingvallavatn	-	-	Planktivorous	PL	Pelagic	24	Not published	Peak in late September – mid-Octobe. Littoral zone (Skúlason et al., 1989)
Iceland	Thingvallavatn	-	-	Piscivorous	Pi	Benthic *Nitella* zone	22	Not published	Mid-September - mid-October. Littoral zone (Skúlason et al., 1989)
Iceland	Mývatn	~2,300 (Einarsson et al., 2004)	4.5	Large generalist	LG	Pelagic/ Benthic	22*	Not published	late October, peaks in November (Guðni Guðbergsson (personal communication))
Iceland	Mývatn	-	-	Small benthic (Krús)	SB	Benthic	30*	Not published	October, November, up to December (Guðni Guðbergsson (personal communication))

* Preliminary assignment

Table S2 Genome assembly data summary

Technology	ENA accession	Read count	Base count (Gb)
HiFi PacBio Sequel IIe	PRJEB76174	8,677,046	157.07
Hi-C Illumina NovaSeq 6000	PRJEB76174	386,000,000	115.0
RNA Illumina NovaSeq 6000	PRJEB76174	467,823,625	140.3
IsoSeq PacBio Sequel IIe	PRJEB76174	7,273,335	23.05

Table S3 Association table matching the 40 chromosome-level scaffolds from *Salvelinus alpinus* (fSalAlp1.1.hap1.cur.20231016) to chromosome-level scaffolds from a possible hybrid between Arctic charr and the Northern Dolly Varden (*S. malma malma*, GCA_002910315.2) using D-GENIES (v1.5.0). Each row lists a scaffold in the query assembly (GCA_002910315.2) and the best matching scaffold in the target assembly (fSalAlp1.1.hap1.cur.20231016) with strand orientation and alignment coordinates.

Query	Target	Strand	Q-length	Q-start	Q-stop	T-length	T-start	T-stop
LG4	1	+	90,115,011	16,205	90,114,916	118,721,705	1,489,695	116,394,216
LG20	2	+	79,996,362	45	79,996,271	114,126,047	351,070	114,123,327
LG18	3	-	72,741,121	2,829	72,741,082	104,245,013	717,794	100,318,516
LG15	4	+	67,329,100	26,255	67,329,018	101,695,431	6,491,007	101,321,349
LG6	5	-	26,274,522	3,725,469	26,274,516	96,462,065	5,917,102	84,633,033
LG8	6	+	54,842,065	9	54,841,694	88,806,950	5,309,520	88,739,146
LG13	7	+	50,975,424	40,692	50,969,248	86,612,117	6,197,812	82,140,713
LG14	8	-	54,096,485	1,566	54,096,482	84,835,329	83,977	77,719,879
LG3	9	+	36,001,405	12,338	35,953,404	67,911,838	14,451,342	65,747,925
LG27	10	-	38,733,064	21,401	38,733,055	67,336,377	10,310,970	57,806,011
LG1	11	+	58,017,395	4,595,352	58,017,368	66,075,642	3,205,685	66,054,031
LG11	12	-	51,124,027	111	51,123,949	63,036,960	7,269,983	62,683,859
LG26	14	-	49,931,436	8	49,931,426	59,249,716	464,143	56,983,757
LG23	15	+	49,632,736	5,924	49,632,670	58,577,525	7,436,261	57,761,919
LG36	16	+	41,232,801	0	41,184,820	57,903,262	89,835	48,039,762
LG17	17	+	41,841,263	47,746	41,768,400	55,992,869	3,131,287	55,518,019

LG16	18	-	42,871,064	2	42,871,055	55,275,544	340,410	53,874,371
LG30	19	+	26,193,892	6,081	26,193,892	54,372,910	7,539,068	45,977,644
LG33	20	+	38,084,510	35	38,084,475	53,120,343	1,400,805	46,090,629
LG2	21	+	43,538,721	294	43,516,696	52,636,049	284,323	52,308,729
LG5	22	+	37,080,635	12,794	37,080,198	48,402,865	702,115	42,234,317
LG22	23	-	37,604,395	8	37,604,369	51,610,363	18,608	51,450,957
LG32	24	-	38,480,802	12,960	38,480,478	50,942,114	9,417,594	50,709,322
LG31	26	-	32,006,513	1,741	31,999,673	49,268,691	8,947,780	49,014,230
LG9	27	+	32,654,316	112	32,652,745	48,381,063	565,603	41,266,040
LG28	28	+	32,734,159	11,425	32,734,155	43,278,249	3,799,949	42,604,607
LG7	29	+	34,303,021	20	34,302,938	46,712,474	561,386	42,282,558
LG19	31	-	38,228,754	45,663	38,228,663	46,280,313	4,381,811	46,272,723
LG10	32	+	22,457,292	36,550	22,457,177	44,767,011	30,308	35,173,697
LG34	33	+	8,958,605	16,087	8,958,562	44,132,330	7,415,860	42,331,747
LG37	34	-	19,546,989	32,242	19,546,946	40,709,877	2	35,199,160
LG35	35	-	21,595,701	66	21,595,695	41,097,330	23,155	30,697,108
LG25	36	-	26,198,113	14	26,198,104	40,659,629	11,597,466	40,558,445
LG21	38	+	6,905,391	12,817	6,903,892	34,184,328	181,603	33,952,954
LG24	39	-	11,432,800	51	11,431,778	33,174,080	10,478,063	32,379,045
LG12	40	+	13,980,584	5	13,971,945	28,249,303	8,508,797	28,237,001

Table S4 Association table matching the 40 chromosome-level scaffolds from *Salvelinus alpinus* (fSalAlp1.1.hap1.cur.20231016) to chromosome-level scaffolds from a selectively bred line of Arctic charr (*Salvelinus alpinus*; GCA_045679555.1) using D-GENIES (v1.5.0). Each row lists a scaffold in the query assembly (GCA_045679555.1) and its best matching scaffold in the target assembly (fSalAlp1.1.hap1.cur.20231016) with strand orientation and alignment coordinates.

Query	Target	Strand	Q-length	Q-start	Q-stop	T-length	T-start	T-stop
34	1	+	27,748,105	5	27,747,346	118,721,705	47,560	109,575,151
9	1	-	87,499,367	23	87,498,368	118,721,705	485,348	118,699,495
1	2	+	120,996,972	14,621	120,995,520	114,126,047	1,091	114,125,963
3	3	-	112,556,904	1,437	112,549,616	104,245,013	36,287	104,226,481
5	4	-	103,520,236	395	103,128,390	101,695,431	32,650	101,223,953
4	5	-	103,748,206	1,923	103,631,648	96,462,065	86,246	96,442,626
7	6	-	89,680,855	2,852	89,680,822	88,806,950	168,773	88,806,948
6	7	+	101,172,370	57	97,852,879	86,612,117	45,608	86,577,420
8	8	-	87,809,997	209	87,809,805	84,835,329	83,868	84,754,325
11	9	+	75,605,943	527,596	75,602,411	67,911,838	120,248	67,862,192
10	10	-	87,060,325	10	86,835,741	67,336,377	84,889	67,328,289
2	11	-	130,982,949	11,760,131	130,977,471	66,075,642	461,122	65,821,590
12	12	-	62,624,571	1,314	62,624,538	63,036,960	33,066	62,684,857
18	13	+	53,682,478	36	53,649,638	59,954,136	2,817,836	58,984,769
15	14	-	58,617,916	1,467	58,617,533	59,249,716	196,467	59,196,072
13	15	-	59,638,343	427,764	59,638,337	58,577,525	132	58,391,758
14	16	+	58,912,418	59,402	58,912,088	57,903,262	4	57,865,958
17	17	-	55,619,826	54	55,541,353	55,992,869	18,071	55,989,966
16	18	-	57,379,347	343	57,379,311	55,275,544	127,896	55,272,529
29	19	+	47,909,558	3,237	47,908,946	54,372,910	73,966	48,215,064
19	20	-	53,380,280	23,703	53,380,126	53,120,343	282,742	52,959,484
20	21	-	53,359,140	185	53,338,537	52,636,049	211,527	52,636,022
24	22	+	51,335,609	18,987	51,335,345	48,402,865	6,084	48,402,780

21	23	-	52,710,984	15,779	52,710,971	51,610,363	387,706	51,610,286
23	24	+	51,468,667	19	51,411,232	50,942,114	341,553	50,894,994
26	26	-	48,586,818	229,505	48,576,725	49,268,691	1,516	49,260,503
25	27	+	49,815,695	6	49,815,172	48,381,063	44,459	48,381,005
27	28	+	48,394,982	2,040	48,394,957	43,278,249	7	43,267,839
28	29	+	48,189,912	1,154	48,182,655	46,712,474	561,376	46,686,769
22	30	+	51,875,803	377,573	51,875,739	46,408,077	26	46,331,006
30	31	+	47,366,167	363,600	47,366,166	46,280,313	6	46,279,817
33	32	+	30,788,372	354,230	30,773,782	44,767,011	14	37,448,283
39	33	-	9,909,249	290	9,908,771	44,132,330	34,413,413	44,131,854
31	34	+	43,559,359	180,081	43,559,353	40,709,877	2	40,660,151
35	35	-	25,484,251	2,223	25,484,229	41,097,330	237	36,859,539
32	36	-	30,828,724	42,806	30,743,341	40,659,629	9,739,861	40,659,624
36	37	+	21,150,175	324,244	21,150,155	36,629,820	201	19,765,334
37	39	-	18,865,345	3	18,815,330	33,174,080	13,814,370	33,167,279
38	40	+	14,612,993	11,134	14,612,989	28,249,303	4,889,483	28,230,473

Table S5 Nucleotide diversity (θ) assessed across the whole genome among Arctic charr morphs and populations. Morph abbreviations for Sirdalsvatnet and Vangsvatnet: Dwaf benthic (DB) and Large pelagic (LP); for Mývatn, Large generalist (LG) and Small benthic (Krús, SB), and Thingvallavatn, Piscivorous (Pi), Planktivorous (PL), Large benthivorous (LB) and Small benthivorous (SB).

Country	Lake	Morph	Mean theta
**Across populations**			
Norway	Sirdalsvatnet	All	0.0022
Norway	Vangsvatnet	All	0.0020
Iceland	Thingvallavatn	All	0.0016
Iceland	Mývatn	All	0.0017
**Across morphs**			
Norway	Sirdalsvatnet	DB	0.0021
Norway	Sirdalsvatnet	LP	0.0022
Norway	Vangsvatnet	DB	0.0021
Norway	Vangsvatnet	LP	0.0021
Iceland	Mývatn	SB	0.0016
Iceland	Mývatn	LG	0.0016
Iceland	Thingvallavatn	PL	0.0014
Iceland	Thingvallavatn	Pi	0.0015
Iceland	Thingvallavatn	LB	0.0014
Iceland	Thingvallavatn	DB	0.0015
Iceland	Thingvallavatn	Benthic	0.0015
Iceland	Thingvallavatn	Pelagic	0.0015

Table S6 Number of scaffolds and SNPs with at least one marker exceeding the Bonferroni-corrected significance threshold ($\alpha = 10^{-3}$), and genome-wide average F*st* for each contrast between Arctic charr morphs across four lakes.

Lake	Contrast*	Fst±s.d.	Number of scaffolds	Number of SNPs	Number of SNPs on unplaced scaffolds
Sirdalsvatnet	DB vs. LP	0.19±0.18	40	103,326	1169
Vangsvatnet	DB vs. LP	0.01±0.006	0	0	0
Mývatn	SB vs. LG	0.03±0.05	20	198	2
Thingvallavatn	SB vs. LB	0.04±0.06	33	2,898	9
Thingvallavatn	SB vs. PL	0.08±0.11	39	9,122	31
Thingvallavatn	SB vs. Pi	0.06±0.08	32	2,377	4
Thingvallavatn	LB vs. PL	0.08±0.10	40	9,861	120
Thingvallavatn	LB vs Pi	0.06±0.07	30	563	5
Thingvallavatn	PL vs. Pi	0.02±0.02	1	1	0
Thingvallavatn	Benthic vs. Pelagic	0.06±0.08	40	24,852	142

*Morph abbreviations for Sirdalsvatnet and Vangsvatnet: Dwarf benthic (DB) and Large pelagic (LP); for Mývatn, Large generalist (LG) and Small benthic (Krús, SB), and Thingvallavatn, Piscivorous (Pi), Planktivorous (PL), Large benthivorous (LB) and Small benthivorous (SB).

Table S7 Linkage disequilibrium (r^2) statistics for outlier SNPs (P <1×10^{-10}) with the tops significant SNPs within every inversion region among Arctic charr morphs from Lake Thingvallavatn.

Putative inversion region: scaffold: start - end (Mb)	Contrast	Top significant SNP (bp)	Mean r^2	SD	Min	Max	N SNPs
4: 75.25-76.13	LB vs SB	75529142	0.47	0.07	0.19	0.82	488
5: 22.30-22.75	LB vs SB	22426871	0.47	0.10	0.16	0.68	408
9: 61.30-62.11	LB vs SB	61471683	0.47	0.08	0.20	0.86	276
17: 32.45-33.20	LB vs SB	32588832	0.42	0.10	0.23	0.71	272
1: 16.30-18.60	Benthic vs Pelagic	17783026	0.40	0.10	0.10	0.95	1413
1: 19.50-22.20	Benthic vs Pelagic	33805357	0.35	0.08	0.15	0.57	786
3: 33.50-35.80	Benthic vs Pelagic	29598356	0.42	0.10	0.19	0.66	486
3: 37.35-40.60	Benthic vs Pelagic	6765722	0.45	0.08	0.20	0.63	461
8: 29.05-29.83	Benthic vs Pelagic	16706990	0.30	0.05	0.17	0.46	258
9: 38.40-40.80	Benthic vs Pelagic	17783026	0.40	0.10	0.10	0.95	1413
14: 6.33-6.87	Benthic vs Pelagic	21352301	0.31	0.08	0.09	0.67	864
40: 16.25-17.01	Benthic vs Pelagic	33805357	0.35	0.08	0.15	0.57	786

Table S8 Gene list for regions within 5 kb upstream and 5 kb downstream of putative inversions distinguishing morphs in Thingvallavatn, including Scaffold 34 (18.36–18.45 Mb), associated with haplotypes distinguishing benthic and pelagic morphs in Mývatn and Thingvallavatn.

(Large excel spreadsheet added separately)

Table S9 Summary of the most differentiated SNPs ($P < 1\times10^{-15}$) for each diagnostic region, including their nearest gene, and relative position to genes (e.g., missense, synonymous, upstream, downstream, or intergenic) as determined by snpEff.

(Large excel spreadsheet added separately)

Table S10 Genotype distribution at four putative inversions showing genetic differentiation between the small and large benthivorous and large benthic morphs present in Thingvallavatn.

Group	Scaffold and region (start-end, Mb)							
	4: 75.25-76.13		5: 22.30-22.75		9: 61.30-62.11		17: 32.45-33.20	
	SB	LB	SB	LB	SB	LB	SB	LB
Homozygous major (small benthivorous)	26	1	26	0	26	1	24	0
Heterozygous	1	9	1	5	1	6	3	5
Homozygous minor (large benthivorous)	0	28	0	33	0	31	0	33
Total	27	38	27	38	27	38	27	38

Table S11 Nucleotide diversity (θ) assessed across the whole genome and at putative inversion regions among Arctic charr morphs from Thingvallavatn homozygous for small and large benthivorous haplotype.

Scaffold: start - end	Haplotype	Mean θ
Genome-wide	Small benthivorous	0.0015
Genome-wide	Large benthivorous	0.0014
4: 75.25-76.13	Small benthivorous	0.0007
	Large benthivorous	0.0005
5: 22.30-22.75	Small benthivorous	0.0006
	Large benthivorous	0.0004
9: 61.30-62.11	Small benthivorous	0.0021
	Large benthivorous	0.0013
17: 32.45-33.20	Small benthivorous	0.0004
	Large benthivorous	0.0004

Table S12 Genotype distribution at eight putative inversions showing genetic differentiation between the benthic (B) and pelagic (P) morphs present in Thingvallavatn.

Group	Scaffold and region (start-end, Mb)															
	1: 16.30-18.60		1: 19.50-22.20		3: 33.50-35.80		3: 37.35-40.60		8: 29.05-29.83		9: 38.40-40.80		14: 6.33-6.87		40: 16.25-17.01	
	B	P	B	P	B	P	B	P	B	P	B	P	B	P	B	P
Homozygous major (benthic)	62	5	31	0	62	6	52	3	22	0	48	2	56	8	26	1
Heterozygous	2	21	29	16	2	25	10	15	30	1	15	17	8	17	30	9
Homozygous minor (pelagic)	0	20	4	30	0	15	2	28	12	45	1	27	0	21	8	36
Total	64	46	64	46	64	46	64	46	64	46	64	46	64	46	64	46

Table S13 Nucleotide diversity (θ) assessed across the whole genome and at putative inversion regions among Arctic charr morphs from Lake Thingvallavatn homozygous for benthic or pelagic haplotype.

Scaffold: start - end (Mb)	Haplotype	Mean pairwise theta
Genome-wide	Benthic	0.0015
Genome-wide	Pelagic	0.0015
1: 16.30-18.60	Benthic	0.0018
	Pelagic	0.0018
1: 19.50-22.20	Benthic	0.0005
	Pelagic	0.0008
3: 33.50-35.80	Benthic	0.0006
	Pelagic	0.0007
3: 37.35-40.60	Benthic	0.0004
	Pelagic	0.0004
8: 29.05-29.83	Benthic	0.0008
	Pelagic	0.0008
9: 38.40-40.80	Benthic	0.0006
	Pelagic	0.0008
14: 6.33-6.87	Benthic	0.0004
	Pelagic	0.0005
40: 16.25-17.01	Benthic	0.0006
	Pelagic	0.0015

Table S14 Genotype distribution at locus 18.36-18.45 Mb of scaffold 34 showing genetic differentiation between morphs homozygous for benthic and pelagic haplotype present in Thingvallavatn and Mývatn.

	Scaffold 34: 18.36-18.45 Mb						
	Mývatn		Thingvallavatn				Total
Group	LG	SB	SB	LB	PL	Pi	
Homozygous major (benthic)	2	10	18	28	0	1	59
Heterozygous	9	3	8	7	0	6	33
Homozygous minor (pelagic)	28	0	1	3	24	15	71
Total	39	13	27	38	24	22	163
